# Hierarchically Structured Deformation‐Sensing Mechanochromic Pigments

**DOI:** 10.1002/advs.202206416

**Published:** 2023-03-19

**Authors:** Jess M. Clough, Cédric Kilchoer, Bodo D. Wilts, Christoph Weder

**Affiliations:** ^1^ Adolphe Merkle Institute University of Fribourg Chemin des Verdiers 4 Fribourg 1700 Switzerland; ^2^ Chemistry and Physics of Materials University of Salzburg Jakob‐Haringer‐Strasse 2a Salzburg 5020 Austria

**Keywords:** mechanochemistry, photonics, pigments, polymers, sensing

## Abstract

Mechanochromic materials alter their color in response to mechanical force and are useful for both fundamental studies and practical applications. Several approaches are used to render polymers mechanochromic, but they generally suffer from limitations in sensing range, capacity to provide quantitative information, and their capability to enable broad and simple implementation. Here, is it reported that these problems can be overcome by combining photonic structures, which alter their reflection upon deformation, with covalent mechanophores, whose spectral properties change upon mechanically induced bond scission, in hierarchically structured mechanochromic pigments. This is achieved by synthesizing microspheres consisting of an elastic polymer with spiropyran‐based cross‐links and non‐close‐packed silica nanoparticles. A strain of less than 1% can be detected in a shift of the reflection band from the photonic structure, while the onset strain for the conversion of the spiropyran into fluorescent merocyanine ranges from 30% to 70%, creating a broad strain detection range. The two responses are tailorable and synergistic, permitting the activation strain for the mechanophore response to be tuned. The mechano‐sensing photonic pigments are demonstrated to be readily incorporated into different polymeric materials of interest and quantitatively probe spatially heterogeneous deformations over a large strain range.

## Graphical abstract

Mechanochromic sensors for polymeric materials with a desirable combination of properties are reported and incorporated in several polymeric matrices. The sensors are easily deployable microparticles, or mechanochromic pigments, whose spectral response is readily monitored during deformation. By combining two mechano‐sensing principles, the pigments visualize and quantify spatially heterogeneous deformation in polymeric materials over a wide strain range.

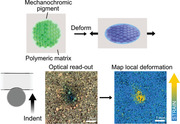

## 1 Introduction

The development of mechanically responsive polymers is currently attracting considerable interest^[^ ^1^, ^2^, ^3^ ^]^ that is driven by the goal to understand how macroscopic forces are transduced to the microscopic, nanoscopic and molecular length scales,^[^ ^4^, ^5^ ^]^ and the desire to create materials that translate mechanical cues into useful responses.^[^ ^6^ ^]^ Mechanochromic polymers, which change their color and/or photoluminescence characteristics upon deformation and permit the visualization and sometimes quantification of stress and/or strain, represent an important subset of this class of materials.^[^ ^6^, ^7^, ^8^, ^9^, ^10^, ^11^ ^]^ Mechanochromic effects permit the monitoring of complex mechanical behaviors and failure processes in polymers and other materials,^[^ ^6^ ^]^ and they are technologically useful for force or strain sensing, structural health monitoring, and many other applications.^[^ ^12^, ^13^, ^14^ ^]^ Mechanochromic sensing schemes can provide information that is difficult or impossible to acquire by other optomechanical detection methods, such as laser speckle imaging,^[^ ^15^ ^]^ digital image correlation,^[^ ^16^, ^17^ ^]^ and photoelasticity,^[^ ^18^ ^]^ including, for example, insights into internal local stresses and the effects of mechanical force at the molecular level. Moreover, the detection of the spectral changes involved in mechanochromic effects is experimentally more accessible than the techniques mentioned above.

Different strategies have been developed to render polymer materials mechanochromic,^[^ ^6^, ^10^, ^14^, ^19^ ^]^ including the release of (latent) dyes from rupturing capsules,^[^ ^20^ ^]^ rearrangements of plasmonic materials,^[^ ^21^ ^]^ the separation of optically active moieties that engage in inter‐^[^ ^22^, ^23^ ^]^ or intramolecular interactions,^[^ ^24^, ^25^, ^26^ ^]^ conformational changes of conjugated polymers^[^ ^27^ ^]^ and small conjugated groups in polymers,^[^ ^28^ ^]^ unit cell changes of photonic nanostructures,^[^ ^12^, ^29^, ^30^, ^31^, ^32^, ^33^, ^34^, ^35^, ^36^, ^37^, ^38^ ^]^ as well as the covalent incorporation of mechanically labile molecular moieties referred to as “mechanophores”.^[^ ^6^, ^10^, ^39^ ^]^ The origin of the color change, the stress and strain ranges in which the optical response occurs, the spatial and strain resolution, the possibility to provide quantitative information about the deformation, and the ease of implementation vary greatly between the different approaches.^[^ ^6^, ^26^, ^34^ ^]^ Indeed, many of these platforms have only been tested in tailor‐made materials.^[^ ^40^ ^]^ For example, structurally colored mechanochromic films have been demonstrated to report their own mechanical deformation, or that of a substrate matrix to which the mechanochromic film was adhered or in which the film was embedded.^[^ ^30^, ^38^, ^40^, ^41^ ^]^ However, these materials are limited in their capacity for sensing local strains in a substrate matrix, as they can only report the deformation at the surface of or at a certain depth within the substrate. While interesting strategies have been explored to produce structurally colored mechanochromic materials in other forms apart from films, for example with 3D‐printing,^[^ ^42^ ^]^ it remains difficult to apply these materials as general sensors for deformation within a range of different polymeric matrices. Moreover, the lack of universally applicable mechanochromic deformation sensors may be one key obstacle that has limited their widespread application in commercial products.

**Figure 1:**
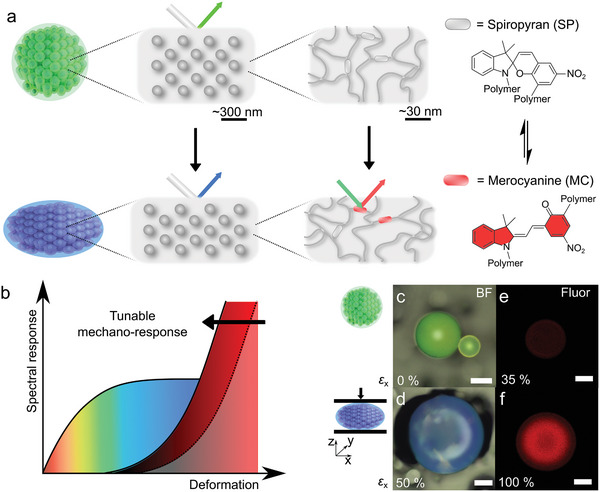
Operating principle of the hierarchically structured mechano‐pigments. a) The mechano‐pigments are comprised of silica nanoparticles that assemble into photonic structures and an elastic polymeric matrix cross‐linked with spiropyran mechanophores. Upon deformation, the periodicity of the photonic assembly changes and at greater strains, the non‐emissive spiropyran is converted into a merocyanine that fluoresces red upon excitation with green light. b) The photonic response is immediate and particularly pronounced at small strains, whereas the activation of the spiropyran mechanophore requires larger deformations and can also be tuned by the composition of the pigment. c–f) Micrographs of a mechano‐pigment whose photonic reflection is green in the absence of force (c) and changes to blue (d) upon compression to 50% strain; even at 35% strain, the spiropyran remains non‐fluorescent (e), whereas at higher strain, the characteristic merocyanine fluorescence is observed (f). Pictures in (c,d) and (e,f) were recorded under brightfield illumination and 530 nm excitation with emission at 650 nm, respectively (scale bars = 50 µm). The mechano‐pigments contain silica particles with a diameter, *d* of 166 nm and silica volume fraction, *φ*(SiO_2_) = 0.35, and a poly(poly(ethylene glycol) phenyl ether acrylate) (p(PEGPEA)) network with a cross‐link density of 0.25 mol% spiropyran and 1 mol% overall.

We here report that different mechanochromic transduction principles and their respective sensing capabilities can be integrated in hierarchically structured “mechano‐pigments,” which are readily deployed in a range of polymeric matrices. These mechano‐sensors are spherical microparticles containing non‐close‐packed silica nanoparticles in an elastic matrix that is cross‐linked with a spiropyran‐based mechanophore (**Figure** **1**a,b).^[^ ^43^, ^44^, ^45^ ^]^ Like pigments found in paints or inks, mechano‐pigments are particulate colorants that are insoluble in the host medium or matrix, which, unlike their traditional counterparts, can also report on the local mechanical properties of the surrounding matrix. While the mechano‐pigments have a diameter of ≈100 µm, the arrangement of the colloidal silica within the microparticle of the mechano‐pigment is correlated on the length scale of ≈200 nm, corresponding to the average nearest neighbor distance between silica colloids. The colloidal array therefore produces a reflection band in the visible wavelength range which shifts at low applied mechanical strains, whereas the spiropyran transforms at much greater deformations to a colored and fluorescent merocyanine (Figure 1c–f).

In contrast to mechanochromic films, mechano‐pigments are of broad, fundamental interest for mechano‐sensing, because their design ensures that they can be readily exploited in any type of matrix, including thermoplastics, thermosets, and composites. Additionally, it is considerably simpler to impart mechanochromism to polymeric materials with mechano‐pigments in comparison with other strategies, as their incorporation requires no chemical modification of the host polymeric matrix. Here, we applied mechano‐pigments to polyethylene, a matrix that is chemically difficult to functionalize. Furthermore, mechano‐pigments offer a desirable combination of sensing features, including continuous, or “grayscale,” deformation‐sensing over three decades of strain with high strain resolution and inherent mechanical and optical tunability. In particular, the incorporation of the mechanophore within the photonic matrix of the mechano‐pigments produces synergies that permit the mechanochromic responses to be tuned, which would be impossible to achieve in systems comprised of the individual components, or where the two components are layered. This system also offers the potential to modify the emission characteristics of fluorescent mechanophores by means of the interaction between the mechanophore's emission and the stop band of the photonic matrix, as previously demonstrated with fluorescent dyes.^[^ ^46^, ^47^, ^48^, ^49^, ^50^, ^51^ ^]^

## 2 Results and Discussion

A scalable and robust approach based on bulk emulsion templating was used to synthesize mechano‐pigments. Mixtures containing silica nanoparticles (refractive index, *n* ≈ 1.46^[^ ^52^ ^]^), monomer (poly(ethylene glycol) phenyl ether acrylate (PEGPEA), *n* ≈ 1.50^[^ ^33^ ^]^), a mechanically inactive cross‐linker (poly(ethylene glycol) diacrylate (PEGDA)) in combination with the spiropyran cross‐linker, and a photo‐initiator (2‐hydroxy‐2‐methylpropiophenone (HMPP)) were emulsified in an aqueous solution of poly(vinyl alcohol) (PVA), which served as a surfactant; photo‐initiated polymerizations fixated the droplets and generated the mechano‐pigments. The colloidal array of the mechano‐pigment reflects light in the visible wavelength range as a result of the periodicity of the silica arrangement, which is on the length scale of 200 nm, and the refractive index contrast between silica and PEGPEA, which at 0.04 is small but sufficient to enable the material to produce a photonic reflection.^[^ ^53^ ^]^ The optical and mechanical properties of the elastic mechano‐pigments are inherently tunable. Different initial photonic colors are obtained by varying the diameter of the silica nanoparticles, *d* and silica volume fraction, *φ*(SiO_2_) of the mechano‐pigments (Figures S2–S6, Supporting Information), while their stiffness can be adjusted by the concentration of the inactive cross‐linker and *φ*(SiO_2_). Unless otherwise noted, the reported experiments were carried out with green mechano‐pigments (peak reflectance at ≈540 nm; *d* = 166 nm, *φ*(SiO_2_) = 0.35, 1 mol% total cross‐link density, including 0.25 mol% spiropyran).

**Figure 2:**
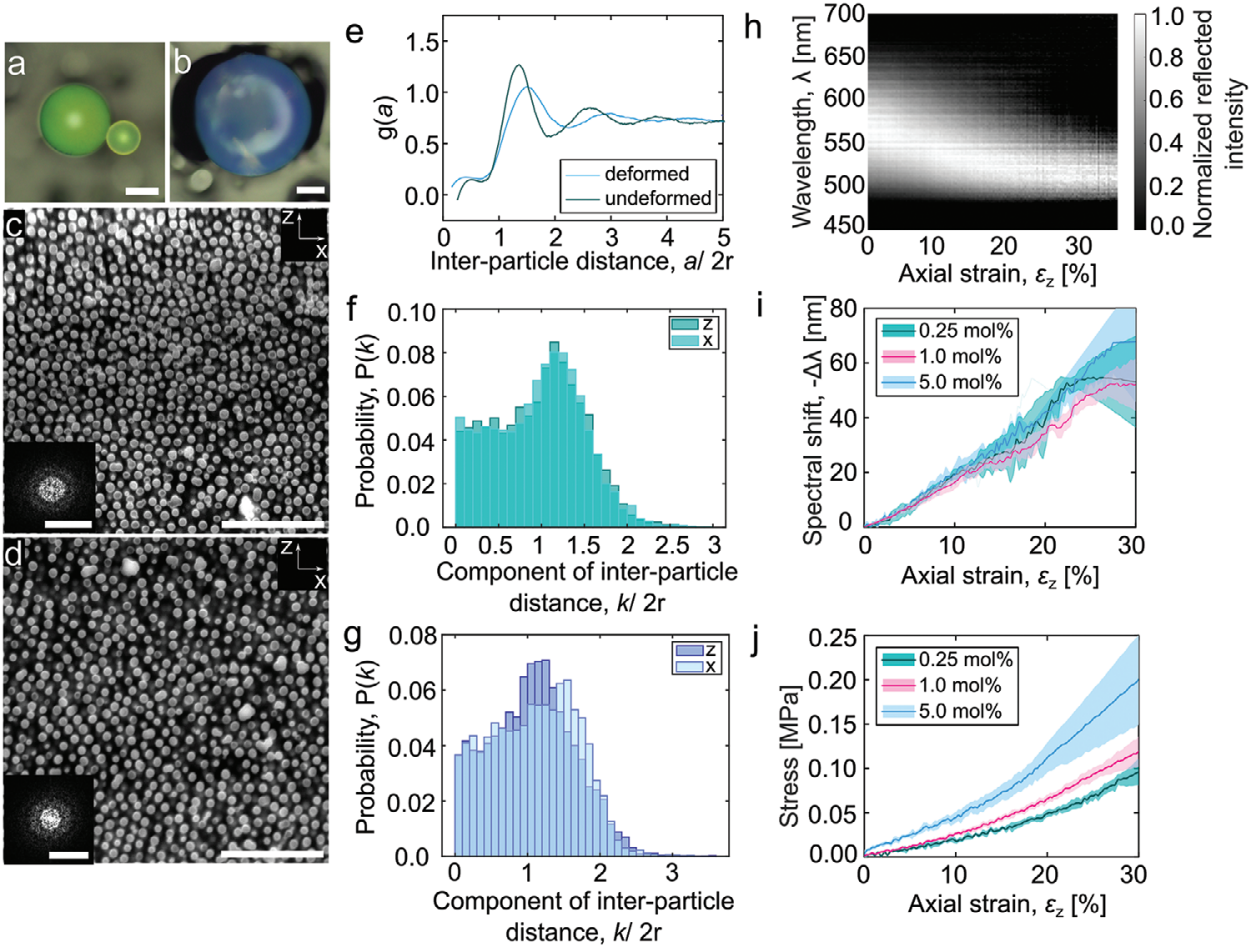
Microstructural changes and photonic mechanochromism of individual mechano‐pigments upon compression. a,b) Light microscopy images of a mechano‐pigment in the as‐prepared state (a) and after compression to 50% strain (b) (scale bar = 50 µm); c,d) SEM images of the cross‐sections of mechano‐pigments in (a,b) (scale bars = 2 µm), with insets showing FFTs (scale bars = 0.02 nm^−1^). e) RDFs of silica nanoparticles calculated from images (c,d). f,g) Histograms of x‐ and z‐components of the inter‐particle distances show deformation‐induced anisotropy in the nanoparticle arrangement after compression (g) compared to the as‐prepared state (f). h) Reflectance changes observed for individual mechano‐pigments (total cross‐link density 0.25 mol%) upon compression. i) Spectral shifts observed for mechano‐pigments with different total cross‐link densities: 0.25 mol% (green), 1 mol% (pink), and 5 mol% (blue) upon compression (with constant spiropyran content of 0.25 mol%). j) Stress‐strain plots of mechano‐pigments with different cross‐link densities. In panels (i,j), solid lines are averages of measurements on at least three mechano‐pigments and shaded areas reflect standard errors. Mechano‐pigment composition (unless otherwise indicated): *d* = 166 nm, *φ*(SiO_2_) = 0.35, 1 mol% total cross‐link density and 0.25 mol% spiropyran.

To investigate their interior microstructure, the cross‐sections of single, green mechano‐pigments were imaged with a focused ion beam (FIB) scanning electron microscope (SEM), in their initial state (**Figure** **2**a,b) and after being compressed to a strain of 50% (Figure 2c,d), which causes some irreversible deformation (vide infra). In the initial state, the interior structure is non‐close‐packed, as intended (Figure 2c, Text S1, Supporting Information) and amorphous at longer inter‐particle distances as indicated by the fast Fourier Transform (FFT) (inset, Figure 2c).^[^ ^54^ ^]^ An analysis of the inter‐particle distances, expressed by a radial distribution function (RDF), shows that the average distance between nearest neighbors is 1.36 *d* (Figure 2e). This value is in good agreement with the distance of 1.3 *d* calculated from the silica: monomer ratio, assuming a local face‐centered cubic packing (Text S2, Table S1, Supporting Information). In comparison to reference mechano‐pigments without the spiropyran (Figure S7, Supporting Information), the addition of the mechanophore appears to reduce the degree of long‐range order moderately, as indicated by the slight broadening of the peaks in the RDF. This may result from the presence of zwitterionic merocyanine in the ethanolic dispersions of silica from which the mechano‐pigments are prepared.^[^ ^55^, ^56^ ^]^ After plastic deformation to a compressive strain of 50%, the reflected color is hypsochromically shifted (Figure 2b), as a result of marked, irreversible microstructural changes (Figure 2d). Histograms of the inter‐particle vector components (Figure 2f,g) and FFTs of the SEM images (insets, Figure 2c,d) demonstrate deformation‐induced anisotropy in the nanoparticle arrangement, that is, a decrease in the average inter‐particle distance along the axis of compression and a concomitant increase in the perpendicular direction. This anisotropy is also reflected in the RDF (Figure 2e), which shows broadened peaks as a result of the increased range of inter‐particle distances.

With this understanding of the deformation‐induced changes in the particle arrangement, the spectral responses of individual mechano‐pigments to compressive force were investigated. To achieve this, we developed a custom set‐up to record the reflectance spectra of individual mechano‐pigments sandwiched between a glass substrate and a silicon wafer, while a compressive strain was applied by movement of the latter (see Supporting Information for further experimental details). As the spherical mechano‐pigments were compressed to form oblate spheroids, the reflectance spectra acquired in the direction of the applied strain (z‐axis) shift hypsochromically, as the average distance between the silica nanoparticles in this direction decreases (Figure 2h, Figure S12, Supporting Information), and the reflected intensity decreases as a result of a decrease in the overall degree of ordering in the photonic structure and a decrease in the effective refractive index on compression (Figure S13, Supporting Information).^[^ ^33^ ^]^ The Young's moduli, *E*, extracted from the compressive stress‐strain curves (in the strain range from 0% to 5%), range from ≈0.50 to 1.50 MPa and increase with the cross‐link density. The *E* values are much lower than the rule‐of‐mixture prediction (25 GPa) based on the volume fraction and moduli of the silica nanoparticles and the polymer matrix in the mechano‐pigment (35 vol% SiO_2_, *E*(SiO_2_) ≈ 70 GPa,^[^ ^57^ ^]^ *E*(polymer) ≈ 0.3 MPa^[^ ^33^ ^]^), indicating that the mechanical behavior of the mechano‐pigments at low strain, where the response is elastic, is largely determined by the polymeric matrix. The elastic nature of the deformation at strains below ≈30% is supported by the reversibility of the spectral changes (Figure S14, Supporting Information). The spectral changes of mechano‐pigments with different cross‐link densities as a function of strain are identical within error (Figure 2i,j) and thus independent of the stiffness of the polymer matrix in the mechano‐pigment. This is consistent with the fact that the response is determined by the applied strain and not stress.

**Figure 3:**
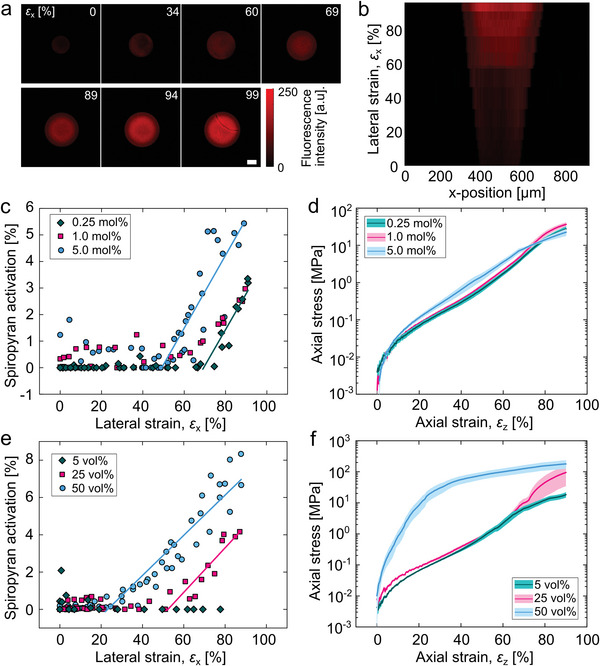
Activation of spiropyran mechanophores in individual mechano‐pigments upon compression. a) Confocal microscopy images of a single mechano‐pigment with a total cross‐link density of 5 mol% (scale bar 50 µm). b) Fluorescence intensity across a central line through the mechano‐pigment as a function of compressive strain. c) Mechano‐activation of spiropyran in individual mechano‐pigments containing polymer networks with three different cross‐link densities (0.25 mol% (green), 1 mol% (pink), and 5 mol% (blue)), calculated by averaging intensity over the central line and normalizing with respect to the fluorescence intensity activated by ultraviolet (UV) irradiation as described in Text S3, Supporting Information, and d) stress‐strain plots for the same mechano‐pigments. e) Increase in spiropyran mechano‐activation with increasing strain for three different *φ*(SiO_2_) (5 vol% (green), 25 vol% (pink), and 50 vol% (blue)), and f) the stress‐strain plots for the same mechano‐pigments. For stress‐strain plots, each curve is an average of at least three mechano‐pigment spheres, with the shaded area corresponding to the standard error in the mean. Mechano‐pigment composition (unless otherwise indicated): *d* = 166 nm, *φ*(SiO_2_) = 0.35, 1 mol% total cross‐link density and 0.25 mol% spiropyran.

In addition to the photonic response, the deformation of the mechano‐pigments can induce the conversion of the colorless spiropyran mechanophores to fluorescent merocyanine (Figure 1e,f). To quantify the changes in fluorescence intensity upon application of compressive strain, individual mechano‐pigments were monitored with confocal microscopy, again in situ under compression. The fluorescence micrographs shown in **Figure** **3**a and line profiles of the fluorescence intensity in Figure 3b exhibit a transition from a strain range in which the mechano‐pigments remain essentially non‐fluorescent to a regime in which the fluorescence response increases with pressure, with an onset of mechano‐activation at ≈50% lateral strain. Control pigments containing molecularly dissolved spiropyran did not show this two‐regime response; instead, the initial weak fluorescence intensity, originating from merocyanine present at the force‐free equilibrium, decreased slightly with increasing strain (Text S3 and Figure S15, Supporting Information).

Significant spatial variations in fluorescence intensity within individual mechano‐pigments indicate the critical role of the microstructure in the activation of spiropyran as mechano‐activation is first observed in the center of the mechano‐pigment (Figure 3a).^[^ ^10^ ^]^ At larger compressive strains, activation becomes more pronounced in a ring around the center. The spatial changes with increasing strain are most likely the result of an increased silica volume fraction in the central region at greater strains, which makes the application of further force to the polymeric matrix in that region increasingly difficult. This is supported by electron micrographs, which show that under high compression, the surfaces that were in contact with the compression plates are concave (Figure S16, Supporting Information). In addition, FIB‐SEM cross‐sections reveal an increased density of silica nanoparticles in the center of the mechano‐pigment with respect to the as‐prepared state (Figure S16, Supporting Information).

We further characterized the high‐strain response of individual mechano‐pigments with different cross‐link densities and *φ*(SiO_2_). Upon increasing the cross‐link density from 0.25 to 5 mol% (with a constant spiropyran content of 0.25 mol%), the increase in stiffness was modest (Figure 3d), but the onset strain for activation was reduced from ≈70% to 50% (Figure 3c). At higher cross‐link densities, the polymer network bears greater average stress, which increases the likelihood that the spiropyran mechanophores experience a sufficiently high force to activate them.^[^ ^58^, ^59^ ^]^

*φ*(SiO_2_) had an even more pronounced effect on the activation of the mechanophore in the mechano‐pigments (Figure 3e). At 5 vol% SiO_2_, no significant spiropyran activation was observed before the mechano‐pigment fractured. Upon increasing the volume fraction from 25 to 50 vol%, the onset point decreased from ≈50% to 30% lateral strain. At lower *φ*(SiO_2_), the markedly different onset strains for spiropyran mechano‐activation, in contrast to the modest differences in the stress‐strain curves between the different mechano‐pigments, seem to suggest that the silica content influences the true strain that the polymer experiences, as a result of the incompressibility of the silica nanoparticles. At greater *φ*(SiO_2_) (50%), the mechano‐pigments have a Young's modulus that is similar to the other mechano‐pigments at low strains (2 MPa up to ≈5% strain), but then become considerably stiffer (Figure 3f). In this case, the mechanical behavior seems to be determined by the nanoparticles coming into contact even at low strains, which in turn induces high local stresses, resulting in increased spiropyran mechano‐activation. Overall, the composite nature of the mechano‐pigment offers broad scope to tune the spiropyran mechano‐response by simple synthetic handles.

To demonstrate quantitative mechano‐sensing in a technologically relevant material with a complex deformation behavior, linear low‐density polyethylene (LLDPE) was used as a matrix.^[^ ^60^, ^61^, ^62^ ^]^ Limited synthetic routes for covalent functionalization make this polymer a difficult candidate for imparting mechanochromic behavior.^[^ ^60^, ^63^ ^]^ In tension, LLDPE exhibits a stress‐strain profile that is comprised of three distinct regimes, that is, elastic deformation (*E* = 150 MPa), neck formation and propagation, and strain hardening.^[^ ^64^ ^]^ These phenomena occur over a wide strain range (≈500%) that cannot fully be captured with other mechanochromic approaches.^[^ ^60^, ^65^ ^]^

**Figure 4:**
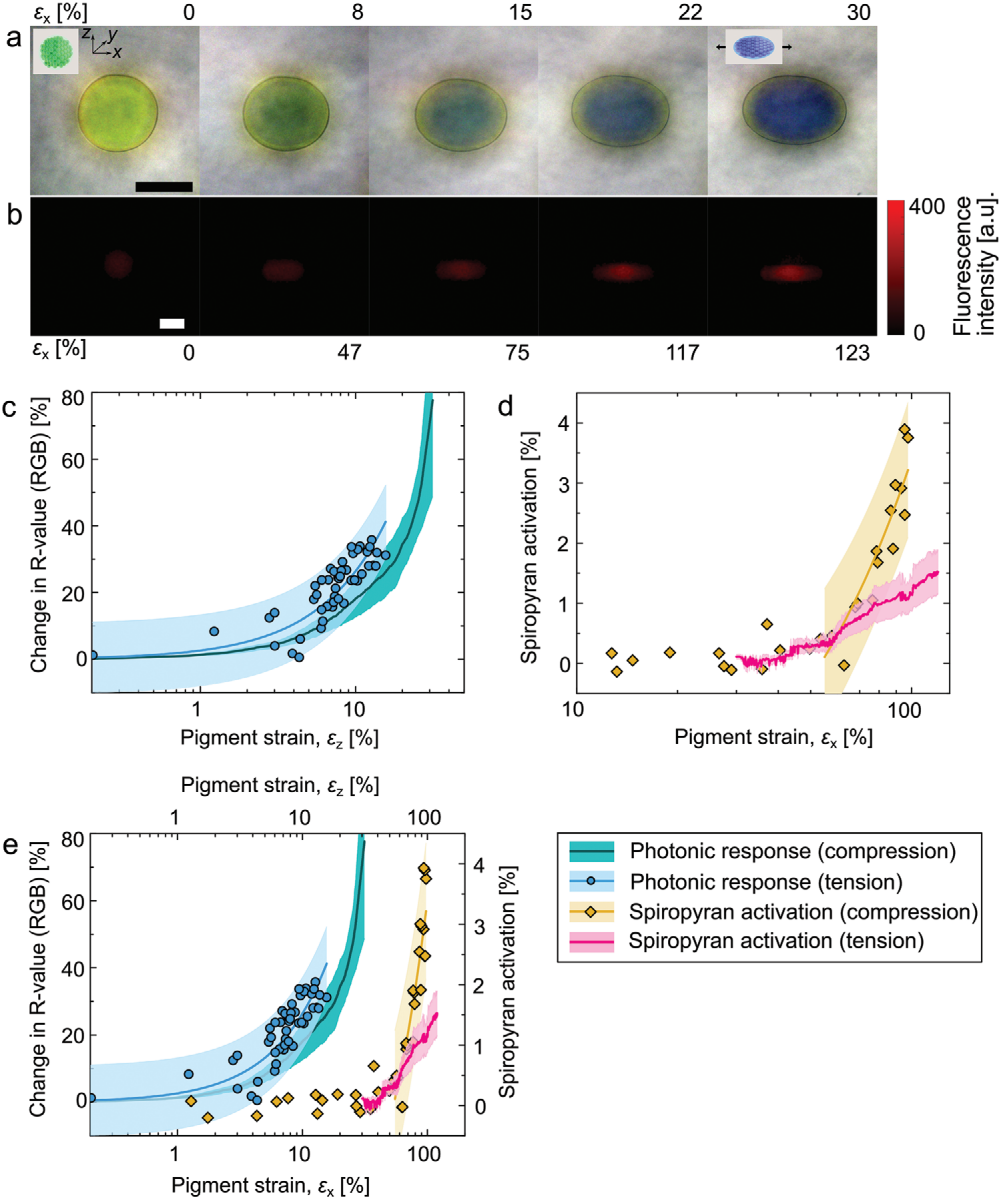
Response of mechano‐pigments in LLDPE. The mechano‐pigments transform into prolate spheroids as the LLDPE matrix is uniaxially strained. a) Light microscopy images documenting the photonic color and shape changes and b) fluorescence microscopy images reflecting an increase of the fluorescence intensity of individual mechano‐pigments as a function of bulk tensile strain (scale bars 100 µm). c) Comparison of the photonic responses of individual mechano‐pigments, that is, not incorporated in a polymeric matrix, under compression (green line, shaded area indicating standard error of the mean) and in an LLDPE matrix under tension (blue line: linear fit to points, with shaded area indicating 95% prediction interval) as a function of local strain. d) Comparison of spiropyran activation in individual mechano‐pigments under compression (yellow line: linear fit to points above activation threshold, shaded area indicating 95% prediction interval) and in an LLDPE matrix under tension (pink line, shaded area indicating standard error in the mean) as a function of local strain. e) Combination of plots (c) and (d) to show strain range covered by mechano‐pigment sensors. The photonic color changes are plotted versus *ε*_z_ and the fluorescence changes versus *ε*_x_. Every data set represents at least three mechano‐pigment spheres. Mechano‐pigment composition: *d* = 166 nm, *φ*(SiO_2_) = 0.35, 1 mol% total cross‐link density and 0.25 mol% spiropyran.

Melt‐mixing permitted the facile incorporation of the mechano‐pigments (0.1 wt%) into LLDPE and the mechanochromic response of compression‐molded films of these composites was studied in uniaxial tensile experiments. The incorporation of mechano‐pigments did not have a significant effect on the Young's modulus of LLDPE (Figure S17, Supporting Information), although the yield stress and strain‐at‐break were slightly reduced at the highest concentration of mechano‐pigments (5 wt%), likely because some mechano‐pigments delaminate from the matrix and/or undergo fracture.^[^ ^66^ ^]^ In the elastic regime, up to the yield point of LLDPE at an elongational strain of ≈30%, the initially spherical mechano‐pigments develop into prolate spheroids and display a hypsochromic structural color shift when viewed along the axis perpendicular to the elongation (**Figure** **4**a,c). The presence of spiropyran within the mechano‐pigment does not change the reflected color from the photonic structure significantly as the spiropyran does not yet activate in this strain range, as evidenced by the lack of change in the fluorescence intensity of the mechano‐pigment. The local elongational strain of the pigments, that is, the extension along their major axis divided by the initial diameter, matches the applied bulk macroscopic strain, indicating that the mechano‐pigments deform concomitantly with the LLDPE matrix (Figure S18, Supporting Information). To compare this response to that of the individual mechano‐pigments in compression (Figure 2i), the strain‐induced structural color change of the individual mechano‐pigments in the film was plotted against the local strain of the mechano‐pigment along the direction in which the pigments experience a compressive strain, which is perpendicular to the macroscopic deformation direction of the film. The similarity of the particles’ mechanochromic response in compression and tension is striking and confirms that the strain applied to the bulk LLDPE matrix is transferred to the mechano‐pigments (Figure 4c).

At ≈30% strain, necking is observed, which induces local strain variations in the matrix.^[^ ^67^, ^68^ ^]^ As the neck passes through the mechano‐pigments, they experience a sudden elongational strain increase and become fluorescent (Figure 4b,d,e). By this extent of deformation, the spectral changes from the photonic structure are no longer detectable. Control experiments with pigments in which the spiropyran isactivated by ultraviolet (UV) irradiation demonstrate that the observed increase in fluorescence is not the result of a change in the optical transmission properties of the polymer film upon necking (Figure S19, Supporting Information). In addition, controls with pigments that contain molecularly dissolved spiropyran do not show a significant fluorescence intensity increase when subjected to tensile strain (Figure S20, Supporting Information). When considering the spiropyran activation as a function of bulk strain, the onset strain for mechano‐activation differs strongly between different mechano‐pigment particles in the same film and depends on the position of the mechano‐pigments relative to the location of the neck (Figure S21, Supporting Information). However, when plotted against the local strain of the mechano‐pigment, as calculated from the dimensions of the mechano‐pigment, these fluorescence curves collapse onto the same “master” curve, demonstrating that the mechano‐pigments act as high‐fidelity reporters of local deformation (Figure 4d,e, Figure S22, Supporting Information). We find that the onset strain for mechano‐activation is slightly lower in compression than in tension and that the intensity changes more significantly with compressive strain than with tensile strain (Figure 4d,e), likely because of the higher stresses experienced under compressive loading.^[^ ^69^ ^]^ Overall, however, the similarity of the responses in tension and compression over three decades in strain demonstrate the broad utility and universality of the mechano‐pigments as a mechano‐sensing platform (Figure 4e).

**Figure 5:**
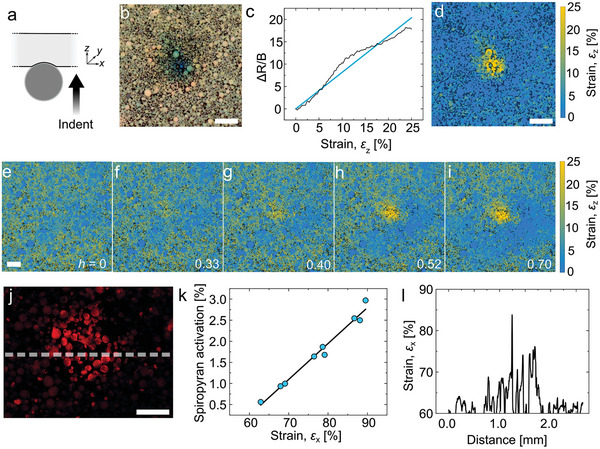
Mapping deformation with mechano‐pigments in PDMS. a) Schematic of indentation geometry. b) Photograph documenting the photonic color changes in mechano‐pigments incorporated in PDMS during deformation (10 wt%) (scale bar = 1.0 mm). c) Calibration curve relating the change in ratio of red to blue values to the strain in the mechano‐pigment (obtained from data in Figure 2i). d) Local strain map of the indented site, obtained from the photograph in (b) using the calibration shown in (c) (scale bar = 1 mm). e–i) Strain maps taken at increasing indentation depths *h*, where *h* = indentation distance/initial thickness of PDMS film (scale bar = 1.0 mm). j) Fluorescence micrograph of a different indented site, showing mechano‐activated fluorescence from the merocyanine (scale bar = 0.5 mm) at an indentation depth *h* ≈ 0.9. k) Calibration curve relating the increase in fluorescence intensity normalized to UV‐activated fluorescence intensity, as described in Text S3, Supporting Information. l) Local compressive strains recorded by mechano‐pigments in the PDMS matrix along the dotted line shown in (j).

Last, the potential of the mechano‐pigments for imaging spatially heterogeneous deformation was demonstrated in an elastomeric polydimethylsiloxane (PDMS) matrix under indentation. This particular matrix was selected for its deformability and optical transparency, although in tension its brittleness^[^ ^70^, ^71^ ^]^ impeded the examination of the mechano‐pigment response in this deformation mode (fracture toughness *K*_Ic_ (PDMS) = 0.2 MPa m^1/2^,^[^ ^15^ ^]^ *K*_c_ (LLDPE) = 7 MPa m^1/2 [^ ^72^ ^]^). When incorporated at a high weight fraction (10 wt%) in PDMS and subjected to indentation with a steel sphere, the mechano‐pigments in the PDMS film exhibited photonic color changes that can be imaged with a simple digital camera under ambient light, as well as fluorescence switch‐on at the indentation site (**Figure** **5**, Text S4 and Figures S24–S28, Supporting Information). Using the spectral calibrations against strain obtained from single mechano‐pigments (Figure 2i), the photonic color changes in the mechano‐pigments (Figure 5b) were converted to local strain values (Figure 5c), generating strain maps of the indented area (Figure 5d). The extent of the indentation area and the peak strain at the center of the indentation site increased with the indentation depth (Figure 5e–i). A similar approach was employed to analyze the fluorescence turn‐on of the spiropyran mechanophores at greater indentation depths (Figure 5j–l). As described in Text S3, Supporting Information, the fluorescence intensity from the mechanically deformed sample was first normalized to the fluorescence intensity obtained upon exposure to UV light. The normalized intensity values were then converted to local strain values by means of the calibration on individual mechano‐pigments (Figures 3c and 5k). Given that it is non‐trivial to relate local strains under indentation to the applied indentation depth,^[^ ^73^ ^]^ these results highlight the powerful simplicity of this mechano‐sensing system to determine local strains from optical signals in a spatially resolved manner.

In summary, this work shows that it is possible to visualize and quantify deformation in polymeric materials over a wide deformation range by combining two mechano‐sensing principles, that is, strain‐induced structural changes of photonic structures and force‐induced activation of fluorescent mechanophores, in easily deployable microparticles whose spectral response is readily monitored in situ during deformation. These mechano‐pigments offer characteristics and properties that are not achievable by the constituent mechano‐sensing approaches individually and their properties can easily be varied. In their current design, the mechano‐pigments would be most suitable as mechano‐sensors in polymeric matrices with *E*(matrix) > *E*(mechano‐pigments) ≈ 1 MPa, covering many commercial organic polymers. We anticipate that changing the nature and architecture of the polymer matrix as well as the mechanophore will allow the tuning of the mechanoresponses of such mechano‐pigments over even wider strain ranges, as well as their application in softer matrices, such as hydrogels.^[^ ^74^ ^]^ This broadly applicable platform opens up new possibilities in developing a better understanding of and predicting failure in polymeric and other soft materials, ranging from 3D printed plastics to tissue cultures.

## 3 Experimental Section

### Preparation of Mechano‐Pigments

To produce 0.5 mL of a silica/PEGPEA mixture, containing silica nanoparticles with *d* = 166 nm, *φ*(SiO_2_) = 0.35, a SP cross‐linker content of 0.25 mol%, and a total cross‐link density of 1 mol%, a suspension of the silica nanoparticles in ethanol with a concentration of 57 mg mL^−1^ was prepared. The density of the SiO_2_ nanoparticles was assumed to be 2 g cm^−3^.^[^ ^75^, ^76^ ^]^ 0.33 mL of the PEGPEA/PEGDA/HMPP mixture and 6.1 mL of the silica dispersion in ethanol were added by a syringe to a glass vial and mixed with a magnetic stirrer. 1.4 mL of a solution of the spiropyran cross‐linker in ethanol (concentration = 1 mg mL^−1^) was added to the dispersion. While stirring, the ethanol was removed under a gentle air flow. The resulting photonic mixture was combined with an aqueous PVA solution (5 mL, concentration = 10 wt%) in a separate small glass vial; the ratio of photonic mixture PVA(aq.) was ≈1:10 v/v. An emulsion was produced by agitating the mixture for 1–2 s with a vortexer (Fisherbrand). The emulsion was then placed in a UV chamber (365 nm, Hoenle LED cube 100) and irradiated with UV light for 30 s at 50% intensity. The dispersion of mechano‐pigment particles was diluted with water (30 mL). The mechano‐pigments were then allowed to sediment and the supernatant was removed with a pipette. The pigments were washed with a 1:1 v/v mixture of ethanol: water, again allowing the pigments to sediment and removing the supernatant, followed by one wash with water and one with ethanol. Finally, the mechano‐pigments were filtered off and dried under vacuum at 50 °C. The mechano‐pigments were obtained in the form of a colored powder. Synthetic details about the preparation of pigments with other compositions can be found in the Supporting Information.

### Compression Molding of Polyethylene Films Containing Mechano‐Pigments

LLDPE pellets were compression‐molded at 110 °C with a pressure of 4 tons for 3 min between two poly(tetrafluoroethylene) sheets with aluminum spacers with a thickness of ≈0.4 mm. The films were subsequently cut into pieces, and dry mechano‐pigments were added between two pieces of LLDPE film. Compression molding with a pressure of 1 ton at 110 °C for 3 min between two Kapton sheets and aluminum spacers with a thickness of ≈0.4 mm produced films of 0.3 mm thickness. Upon removal from the hot press, the films were immediately quenched by immersion in an ice water bath.

### Scanning Electron Microscopy

SEM of the surfaces of the mechano‐pigments and of the silica microspheres was performed on a Tescan Mira3 LM Field Emission microscope. The pigments were cross‐sectioned using a FIB, and their interior microstructure was imaged with a FEI Scios 2 dual‐beam SEM (Ga^+^ ion column and a field‐emission electron gun). Several detectors (T1 or T2) and imaging voltage (2 or 5 kV) were used. Pigments were mounted directly on aluminum stubs with conductive carbon tape, and the silica microspheres were deposited on a silicon (100) wafer, which was then mounted on the aluminum stub. The samples were then coated with a 2.5 nm thick layer of Au using a sputter coater (Cressington 208HR, Cressington Scientific Instruments) to prevent charging.

### Fluorescence Microscopy

Fluorescence microscopy images were acquired at 5× or 20× magnification using an Olympus BX51 microscope equipped with an Olympus DP72 high‐resolution camera. The samples were imaged in reflectance mode using an X‐Cite Series 120‐Q Mercury vapor short arc lamp as the excitation source (brightfield and *λ*_ex_ = 560 nm achieved with a narrow‐band filter). A standard white diffuser was used as a white reference sample.

### Compression of Individual Pigment Particles

Deformation was applied at a strain rate of 0.25–0.5% s^−1^ (indenter velocity 0.5 µm s^−1^) using a motorized actuator (MTS50E/M, Thorlabs with bidirectional repeatability ±0.8 µm) controlled via a computer interface (Kinesis software). Load data were obtained from a load cell (LSB205, maximum load capacity 8.90 N, FUTEK). Images of the pigment prior to and under compression were acquired with a microscope (further details on the in situ optical characterization are provided below). The set‐up was installed on an anti‐vibration table. Individual pigments were selected manually with a pipette or the tip of a micro‐spatula and deposited onto a glass plate with a thickness of 3 mm. The compression tester consisted of a piece of Si (100) wafer adhered to a small screw, which was mounted in the load cell. The initial pigment diameter was obtained from micrographs taken prior to compression. To record the reflectance spectra in situ, the pigments were continuously compressed from above. The indentation experiments were initiated with the indenter slightly above the top of the pigment to allow the indenter to reach a steady‐state velocity before touching the pigment, and terminated at 80% compressive axial strain. To record confocal images in situ, the compressive motion was paused at a given strain to record the images.

### In Situ Reflectance Microscopy

Microspectroscopy during compression experiments was performed using a custom‐built inverted light microscope and a CMOS camera (Blackfly S BFS‐U3‐200S6C‐C, FLIR Integrated Imaging Solutions Inc.). A high‐power Xenon light source (HPX‐2000, Ocean Optics) was used as a light source for all measurements. Dry pigments were deposited on a thick glass slide (3 mm thickness) and positioned below the indenter; the pigments were imaged from below the glass slide in reflection in bright field. The spectral data were collected in reflection mode (bright field) using a 50× objective (BD Plan Apo, Mitutoyo, NA = 0.55). An optical fiber (Ocean Optics, 230 µm core size) was positioned confocal to the image plane, with the other end of the fiber coupled to a diode‐array spectrometer (FLAME‐T‐XR1‐ES, Ocean Insight) in order to measure the spectral reflectance of the pigments. Normalization of the reflectance spectra was performed against a silver mirror (Thorlabs, PF10‐03‐P01, avg. reflectance >97.5% for *λ* = 450–2000 nm). The spectra were recorded continuously (1 spectrum s^−1^) for the duration of the experiment, without refocusing. Spectral changes were similar when the pigments were observed upon stopping the indenter and subsequent refocusing.

### In Situ Confocal Microscopy

Imaging was performed with an inverted Nikon Eclipse TE2000‐U microscope using an excitation laser of 543 nm (Melles Griot) and a 10× objective (Plan, Nikon, NA = 0.25). For each compression step, the imaging plane was refocused manually to ensure that the edges of the pigment were in focus at its largest diameter. Brightfield micrographs were acquired with a digital camera (Evolution MP, Media Cybernetics).

### Tensile Testing

Uniaxial tensile tests were carried out with rectangular samples with dimensions of 4 × 0.3 mm (width × thickness) that were cut from compression‐molded films. The distance between the clamps at zero strain, which defined the initial length of the sample, was 18 mm. A pre‐load of ≈0.1 N was applied before starting the tensile tests. Stress‐strain data collected during in situ microscopic, optical, and fluorescence imaging were recorded at a strain rate of 0.56–0.57% s^−1^ (velocity of clamps 0.1 mm s^−1^) using a Linkam TST350 microtensile stage that was equipped with a 20 N load cell and controlled by accompanying Linksys32 software. The experiments were conducted under ambient conditions (*T* = 23—24 °C).

### Indentation of PDMS Films Containing Mechano‐Pigments

The same set‐up and experimental parameters were used for the indentation experiments as for the compression experiments described above, except the Si (100) wafer was replaced with a steel sphere (diameter 5 mm). The PDMS film (thickness 0.8 mm) was prepared on a glass slide, mounted on the indentation stage and indented from below, while being imaged from above with a camera (Nikon D7100) to record the photonic color changes in the pigments. To record the spiropyran activation, the film was indented, then imaged post indentation with a fluorescence microscope (Olympus BX51).

### Spectral and Image Analysis

Spectra, images, and videos were processed and analyzed in ImageJ (Fiji) and MATLAB (2019b). In particular, the following MATLAB scripts were used to analyze specific microstructural and mechanochromic changes. To generate the radial distribution functions and histograms in Figure 2, the inter‐particle distances were obtained from SEM images with the imfindcircles algorithm, which uses a circular Hough transform. For the analysis of the reflectance spectra in Figure 2, a linear baseline was fitted to the spectra and subtracted from the intensity signal; a Gaussian was then fitted to each baseline‐corrected spectrum, and the wavelength of maximum intensity was taken from the fitted curve and plotted against the strain applied to the pigment. Similar results were obtained using the center of gravity of the baseline‐corrected measured spectra. The confocal images showing the mechano‐activated fluorescence intensity in single pigments, shown in Figure 3, were first corrected for the background intensity and the slight intensity differences arising from the change in focal height between frames. Line profiles of the fluorescence intensity were taken across the central five rows of pixels and averaged; the average values of these intensity profiles were then taken and plotted against pigment strain. For the color analysis of pigments in LLDPE from RGB color images, the pigment was defined manually in each frame as an elliptical region of interest, within which the average RGB values were calculated. To compare the color changes in LLDPE films under tension with those recorded for the free pigment particles under compression, the spectra obtained for the latter were converted to RGB values using the CIE 1931 color space. The microscope images showing fluorescence from pigments in LLDPE were corrected for the uneven illumination across the field of view. The threshold intensity for activation was defined in each frame by a triangle thresholding algorithm.

## Conflict of Interest

The authors declare no conflict of interest.

## Supporting information

Supporting Information

## Data Availability

The data that support the findings of this study are openly available in Hierarchically Structured Deformation‐Sensing Mechanochromic Pigments at https://doi.org/10.5281/zenodo.6834271, reference number 220714.
