# Supplementary material for: Hierarchically Structured Deformation‐Sensing Mechanochromic Pigments
Source: Adv Sci (Weinh). 2023 Mar 19;10(13):2206416. doi: 10.1002/advs.202206416 (PMC10161078; doi:10.1002/advs.202206416)
Supplement: Supplementary file 1 — Supporting Information [file ADVS-10-2206416-s001.pdf]

ADVANCED SCIENCE
Open Access

Supporting Information

for *Adv. Sci.*, DOI 10.1002/advs.202206416

Hierarchically Structured Deformation-Sensing Mechanochromic Pigments

Jess M. Clough, Cédric Kilchoer, Bodo D. Wilts and Christoph Weder*

Supporting Information

Hierarchically Structured Deformation-Sensing Mechanochromic Pigments

Jess M. Clough,[1,*] Cédric Kilchoer,[1] Bodo D. Wilts,[1,2] Christoph Weder[1]

[1]Adolphe Merkle Institute, Chemin des Verdiers 4, 1700 Fribourg, Switzerland

[2]Chemistry and Physics of Materials, University of Salzburg, Jakob-Haringer-Str. 2a, 5020 Salzburg, Austria.

*Email: jessica.clough@unifr.ch

Contents

Materials and Instrumentation 3
Methods and Procedures 5
Supporting Figures 9
References 27

Materials and Instrumentation

Materials. Poly(ethylene glycol) phenyl ether acrylate with a number-average molecular weight (M_n) of 324 g/mol (PEGPEA), poly(ethylene glycol) diacrylate with M_n = 575 g/mol (PEGDA), 2,3,3-trimethylindolenine, hydrobromic acid (48%), poly(vinyl alcohol) with weight-average molecular weight (M_w) = 13,000-23,000 g/mol, 87-89% hydrolyzed) (PVA), 2-dimethyl aminopyridine, methacrylic anhydride, tetraethyl orthosilicate (TEOS), 28-30% ammonium hydroxide and polyethylene (LLDPE, linear low-density, melt index 1.0 g / 10 min (190°C/2.16kg)) were purchased from Sigma Aldrich. Sylgard 184 was purchased from Distrelec Group AG. 2-Hydroxy-5-nitro-m-anisaldehyde, 2-iodoethanol and 2-hydroxy-2-methyl-1-phenyl-1-propane photo-initiator (HMPP) were purchased from TCI. PEGPEA and PEGDA were passed over a short alumina column before use; all other chemicals were used as received. Solvents were purchased from Fisherbrand and used as received. Silica nanoparticles were purchased from Angstrom Spheres (supplier stated size 100 nm diameter) and used as seeds for Stöber synthesis. Water was distilled and deionized to 18.3 Ω cm (Milli-Q). The spiropyran cross-linker (SP) was synthesized following literature procedures and was characterized by NMR spectroscopy.[1,2] No unexpected or unusually high safety hazards were encountered in any of the described procedures.

Nuclear magnetic resonance (NMR) spectroscopy. NMR spectroscopy was performed on a Bruker Avance DPX 400 spectrometer at 297.2 K using frequencies of 400.19 MHz for ^{1}H nuclei and 100.63 MHz for ^{13}C nuclei. Spectra were calibrated to the residual solvent peak of $CDCl_3$. Spectra were evaluated in MestReNova (v 12.0) and all chemical shifts (δ) are reported in parts per million (ppm) relative to tetramethyl silane with coupling constants in Hz (multiplicity: s = singlet, d = doublet, dd = double doublet, t = triplet, m = multiplet, br = broad signal).

Thermogravimetric analysis (TGA). Thermogravimetric analysis (TGA) was conducted on a Mettler Toledo TGA/DSC 1 STAR system from 25 to 550 °C in air with a heating rate of 10 °C min^{-1}.

Dynamic mechanical analysis (DMA). Dynamic mechanical analysis (DMA) was conducted on a TA Instruments DMA Q800. The compression test was conducted with indenter velocity 0.023 mm min^{-1}, static force ramp 0.1 N min^{-1}.

Scanning electron microscopy (SEM). Scanning electron microscopy (SEM) of the surfaces of the mechano-pigments and of the silica microspheres was performed on a Tescan Mira3 LM Field Emission microscope. The pigments were cross-sectioned using a focused ion beam (FIB), and their interior microstructure was imaged with a FEI Scios 2 dual-beam SEM (Ga+ ion column and a field-emission electron gun). Several detectors (T1 or T2) and imaging voltage (2 or 5 kV) were used. Pigments were mounted directly on aluminum stubs with conductive carbon tape, and the silica microspheres were deposited on a silicon (100) wafer, which was then mounted on the aluminum stub. The samples were then coated with a 2.5 nm thick layer of Au using a sputter coater (Cressington 208HR, Cressington

Scientific Instruments) to prevent charging. Electron micrographs obtained from the FIB-SEM were corrected for the tilt of the stage (52°).

Photographs. Photographs and videos of samples were recorded with a Nikon D7100 digital camera equipped with an AF-S DX Zoom-NIKKOR 18-135mm lens (f/3.5-5.6G IF-ED) in manual focus mode under ambient light.

Fluorescence microscopy. Fluorescence microscopy images were acquired at 5x or 20x magnification using an Olympus BX51 microscope equipped with an Olympus DP72 high-resolution camera. The samples were imaged in reflectance mode using an X-Cite Series 120-Q Mercury vapor short arc lamp as the excitation source (brightfield and λ_{ex} = 560 nm). A standard white diffuser was used as a white reference sample.

Microscopic reflectance spectroscopy. Microspectroscopy was performed with a Zeiss Axio Scope.A1 polarized light microscope, connected to a Point Grey GS3-U3-28S5C-C CCD camera (FLIR Integrated Imaging Solutions Inc.). For all measurements, the samples were imaged in reflection mode (bright field) with Koehler illumination, using a halogen light source (Zeiss HAL100). Dry pigments, or pigment-containing PDMS or LLDPE were deposited on a glass slide and imaged in reflection in bright field. To measure the spectral reflectance of the pigments, an optical fiber (Ocean Optics, 230 µm core size) was positioned confocal to the image plane, with the other end of the fiber coupled to a diode-array spectrometer (FLAME-T-XR1-ES, Ocean Insight). Normalization of the reflectance spectra was performed against a silver mirror (Thorlabs, PF10-03-P01, avg. reflectance >97.5% for λ = 450–2000 nm). The spectra were collected using a 20x objective (Zeiss EC Epiplan-Apochromat, NA = 0.6).

Compression of individual pigment particles. Deformation was applied at a strain rate of 0.25-0.5 % s-1 (indenter velocity 0.5 µm s-1) using a motorized actuator (MTS50E/M, Thorlabs with bidirectional repeatability ±0.8 µm) controlled via a computer interface (Kinesis software). Load data were obtained from a load cell (LSB205, maximum load capacity 8.90 N, FUTEK). Images of the pigment prior to and under compression were acquired with a microscope (further details on the in-situ optical characterization are provided below). The set-up was installed on an anti-vibration table. Individual pigments were selected manually with a pipette or the tip of a micro-spatula and deposited onto a glass plate with a thickness of 3 mm. The compression tester consisted of a piece of Si (100) wafer adhered to a small screw, which was mounted in the load cell. The initial pigment diameter was obtained from micrographs taken prior to compression. To record the reflectance spectra in situ, the pigments were continuously compressed from above. The indentation experiments were initiated with the indenter slightly above the top of the pigment to allow the indenter to reach a steady-state velocity before touching the pigment, and terminated at 80% compressive axial strain. To record confocal images in situ, the compressive motion was paused at a given strain to record the images.

In situ reflectance microscopy. Microspectroscopy during compression experiments was performed using a custom-built inverted light microscope and a CMOS camera (Blackfly S BFS-U3-200S6C-C,

FLIR Integrated Imaging Solutions Inc.). A high-power Xenon light source (HPX-2000, Ocean Optics) was used as a light source for all measurements. Dry pigments were deposited on a thick glass slide (3 mm thickness) and positioned below the indenter; the pigments were imaged from below the glass slide in reflection in bright field. The spectral data were collected in reflection mode (bright field) using a 50x objective (BD Plan Apo, Mitutoyo, NA = 0.55). An optical fiber (Ocean Optics, 230 µm core size) was positioned confocal to the image plane, with the other end of the fiber coupled to a diode-array spectrometer (FLAME-T-XR1-ES, Ocean Insight) in order to measure the spectral reflectance of the pigments. Normalization of the reflectance spectra was performed against a silver mirror (Thorlabs, PF10-03-P01, avg. reflectance >97.5% for λ = 450–2000 nm). The spectra were recorded continuously (1 spectrum/s) for the duration of the experiment, without refocusing. Spectral changes were similar when the pigments were observed upon stopping the indenter and subsequent refocusing.

In situ confocal microscopy. Images were acquired with an inverted Nikon Eclipse TE2000-U microscope using an excitation laser of 543 nm (Melles Griot) and a 10x objective (Plan, Nikon, NA = 0.25). For each compression step, the imaging plane was refocused manually to ensure that the edges of the pigment were in focus at its largest diameter (confocal slice thickness ~30 µm). Brightfield micrographs were acquired with a digital camera (Evolution MP, Media Cybernetics).

Tensile testing. Uniaxial tensile tests were carried out with rectangular samples with dimensions of 4 × 0.3 mm (width × thickness) that were cut from compression-molded films. The distance between the clamps at zero strain, which defined the initial length of the sample, was 18 mm. A pre-load of approximately 0.1 N was applied before starting the tensile tests. Stress-strain data collected during in-situ microscopic optical and fluorescence imaging were recorded at a strain rate of 0.56-0.57% s^{-1} (velocity of clamps 0.1 mm s^{-1}) using a Linkam TST350 microtensile stage that was equipped with a 20 N load cell and controlled by accompanying Linksys32 software. The experiments were conducted under ambient conditions (T = 23-24 °C).

Indentation of PDMS films containing mechano-pigments. The same set-up and experimental parameters were used for the indentation experiments as for the compression experiments described above, except the Si (100) wafer was replaced with a steel sphere (diameter 5 mm). The PDMS film (thickness 0.8 mm) was prepared on a glass slide, mounted on the indentation stage and indented from below, while being imaged from above with a camera (Nikon D7100) to record the photonic color changes in the pigments. To record the spiropyran activation, the film was indented, then imaged post mortem with a fluorescence microscope (Olympus BX51).

Methods and Procedures

Synthesis of silica particles. An aqueous dispersion of seed nanoparticles was first prepared by sonicating silica seed nanoparticles (5 g) in ethanol (100 mL) in a 250 mL beaker, first in a bath sonicator

(Huber) for 1 h, then at 40-50% maximum amplitude with a horn sonicator for 2 h (Branson Digital Sonifier, 1 s on, 2 s off). 10 mL of the aqueous silica seed dispersion, ethanol (150 mL), water (35 mL) and NH_4OH (30% aqueous solution, 5 mL) were added to a 500 mL round bottom flask, with a magnetic stir bar. TEOS was added via a syringe pump at 0.8 mL/h, stirring at 400 rpm. The reaction was left to stir for 18 h at room temperature. The dispersion was centrifuged, the supernatant replaced with fresh ethanol and the particles were redispersed in a bath sonicator; this process was then repeated twice. The particles were then dried for 18 h at 70 °C and redispersed in ethanol at a concentration of 50 mg/ mL. The diameters and polydispersity of the silica particles thus made were analyzed from SEM images (Supporting Figure 2 and 3, Table 2).

Preparation of mechano-pigments. Three photonic compositions were studied, with the following parameters (Table 1). The standard *matrix composition* for each photonic composition involved a cross-link density of 1 mol%, including a spiropyran content of 0.25 mol%. For photonic composition **2**, the volume fraction of silica and cross-link density of the matrix was also varied (main article, Figure 2-4). Unless otherwise noted, experiments are reported with particles according to photonic composition **2**, $\varphi(SiO_2) = 0.35$ and an overall cross-link density of 1 mol%, including 0.25 mol% spiropyran.

Table S1. Photonic compositions studied. Errors in the silica particle diameter are standard errors in the mean, calculated from the analysis on three separate SEM images.

Photonic composition number	Silica particle diameter/nm	Volume fraction of silica in photonic mixture, $\varphi(SiO_2)$
**1**	154.3 ± 3.5	0.35
**2**	165.6 ± 4.7	0.35
**3**	197.7 ± 3.3	0.40

To produce 0.5 mL of a silica/PEGPEA mixture, containing silica nanoparticles with a diameter = 163 nm, a silica volume fraction ($\varphi(SiO_2)$) of 0.35, a SP cross-linker content of 0.25 mol%, and a total cross-link density of 1 mol%, a suspension of the silica nanoparticles in ethanol with a concentration of 57 mg/mL was prepared. The density of the SiO_2 nanoparticles was assumed to be 2 g/cm^3.[3–5] 0.33 mL of the PEGPEA/PEGDA/HMPP mixture and 6.1 mL of the silica dispersion in ethanol were added by a syringe to a glass vial and mixed with a magnetic stirrer. 1.4 mL of a solution of the spiropyran cross-linker in ethanol (concentration = 1 mg/mL) was added to the dispersion. While stirring, the ethanol was removed under a gentle air flow. The resulting photonic mixture was combined with an aqueous PVA solution (5 mL, concentration = 10 wt%) in a separate small glass vial; the ratio of photonic mixture

PVA(aq.) was ca. 1:10 v/v. An emulsion was produced by agitating the mixture for 1-2 seconds with a vortexer (Fisherbrand). The emulsion was then placed in a UV chamber (365 nm, Hoenle LED cube 100) and irradiated with UV light for 30 s at 50% intensity. The dispersion of mechano-pigment particles was diluted with water (30 mL). The mechano-pigments were then allowed to sediment and the supernatant was removed with a pipette. The pigments were washed with a 1:1 v/v mixture of ethanol: water, again allowing the pigments to sediment and removing the supernatant, followed by one wash with water and one with ethanol. Finally, the mechano-pigments were filtered off and dried under vacuum at 50 °C. The mechano-pigments were obtained in the form of a colored powder. Other compositions were made using the same protocol. For particles with composition **1**, the following quantities were used: 0.33 mL of the PEGPEA/PEGDA/HMPP mixture and 10.1 mL of the silica dispersion in ethanol (concentration = 34.7 mg/mL). For particles with composition **3**, the following quantities were used: 0.33 mL of the PEGPEA/PEGDA/HMPP mixture and 15.1 mL of the silica dispersion in ethanol (concentration = 26.5 mg/mL). To prepare larger samples for DMA measurements, a mold was prepared from PDMS (2 g, Sylgard 184 1:10 curing agent: base, curing at 65 ºC) using a glass tube 1 mm in diameter, fixed in place in a plastic petri dish (5 cm in diameter) with double-sided tape. The glass tube was removed, and the mold filled with the photonic mixture consisting of silica particles, PEGPEA, PEGDA and HMPP. The mixture was then polymerized under UV light as described above, and the sample was cut from the mold.

Compression molding of poly(ethylene) films containing mechano-pigments. LLDPE pellets were compression-molded at 110 °C with a pressure of 4 tons for 3 min between two poly(tetrafluoro ethylene) (PTFE) sheets with aluminum spacers with a thickness of ca. 0.4 mm. The films were subsequently cut into pieces, and dry mechano-pigments were added between two pieces of LLDPE film. Compression molding with a pressure of 1 ton at 110 °C for 3 min between two Kapton sheets and aluminum spacers with a thickness of ca. 0.4 mm produced films of thickness 0.3 mm. Upon removal from the hot press, the films were immediately quenched by immersion in an ice water bath.

Preparation of PDMS films containing mechano-pigments. Sylgard 184 (0.45 g, 1:10 curing agent: base) was prepared in a 15 mL glass vial by mixing vigorously with a spatula for 2-3 minutes, then vortexing for a minute. 50 mg of dry pigments were then added to this mixture, which was then mixed with a spatula for a further minute and vortexed for a minute. The mixture was spread over a 2.5 x 5 cm area on a glass microscope slide (cleaned by washing with IPA, water then IPA and dried under an air flow), and the slide transferred to a desiccator and placed under vacuum for 30 minutes at room temperature. The slide was then placed in an oven at 65 ºC in air and the mixture cured for 18 hours.

Spectral and image analysis. Spectra, images and videos were processed and analyzed in ImageJ (Fiji) and MATLAB (2019b). In particular, the following MATLAB scripts were used to analyze specific microstructural and mechanochromic changes. To generate the radial distribution functions and histograms in Figure 2, the SEM images were corrected for the angle of the FIB-SEM stage (52º) and

the inter-particle distances were obtained with the imfindcircles algorithm, which uses a circular Hough transform. For the analysis of the reflectance spectra in Figure 2 in the main manuscript, a linear baseline was fitted to the spectra and subtracted from the intensity signal; a Gaussian was then fitted to each baseline-corrected spectrum, and the wavelength of maximum intensity was taken from the fitted curve and plotted against the strain of the pigment. Similar results were obtained using the center of gravity of the baseline-corrected measured spectra. The confocal images showing the mechano-activated fluorescence intensity in single pigments, depicted in Figure 3 in the main manuscript, were first corrected for the background intensity and the slight intensity differences arising from the change in focal height between frames. Line profiles of the fluorescence intensity were taken across the central five rows of pixels and averaged; the average values of these intensity profiles were then taken and plotted against pigment strain. For the color analysis of pigments in LLDPE from RGB color images, the pigment was defined manually in each frame as an elliptical region of interest, within which the average RGB values were calculated. To compare the photonic color changes in LLDPE under tension with those in the absence of a matrix under compression, the spectra obtained for the latter were converted to RGB values using the CIE 1931 color space. The movies showing fluorescence from pigments in LLDPE were corrected for the uneven illumination across the field of view. The threshold intensity for activation was defined in each frame by a triangle thresholding algorithm.

Synthesis and characterization of spiropyran dimethacrylate cross-linker. Literature synthetic procedures were followed[1,2] and the product was characterized by ^{1}H NMR spectroscopy.

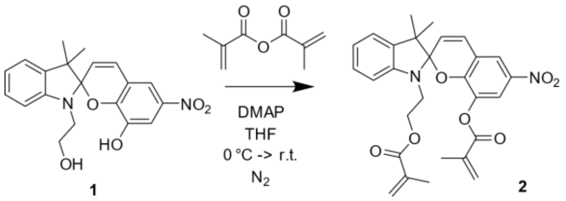

^{1}H NMR (400 MHz, $CDCl_3$) δ: 7.88 (d, *J* = 2.6 Hz, 1H, *H*-Ar), 7.84 (d, *J* = 2.6 Hz, 1H, *H*-Ar), 7.05 (td, *J* = 7.7, 1.3 Hz, 1H, *H*-Ar), 6.94 (m, 1H, CH_2=CCH_3), 6.9 (d, *J* = 10.4 Hz, 1H, C*H*=CH-Ar), 6.76 (td, *J* = 7.4, 0.9 Hz, 1H, *H*-Ar), 6.56 (d, *J* = 7.8 Hz, 1H, *H*-Ar), 6.00 (m, 1H, CH_2=CCH_3), 5.89 (d, *J* = 10.4 Hz, 1H, CH=C*H*-Ar), 5.81 (m, 1H, *H*-Ar), 5.49 (q, *J* = 1.5 Hz, 1H, CH_2=CCH_3), 5.31 (q, *J* = 1.5 Hz, 1H, CH_2=CCH_3), 4.19 (t, *J* = 6.2 Hz, 2H, OCH_2CH_2), 3.29 (t, *J* = 6.2 Hz, 2H, OCH_2CH_2), 1.85 (s, 3H, CH_2=CCH_3), 1.55 (s, 3H, CH_2=CCH_3), 1.18 (s, 3H, CH_3), 1.11 (s, 3H, CH_3).

Supporting Figures

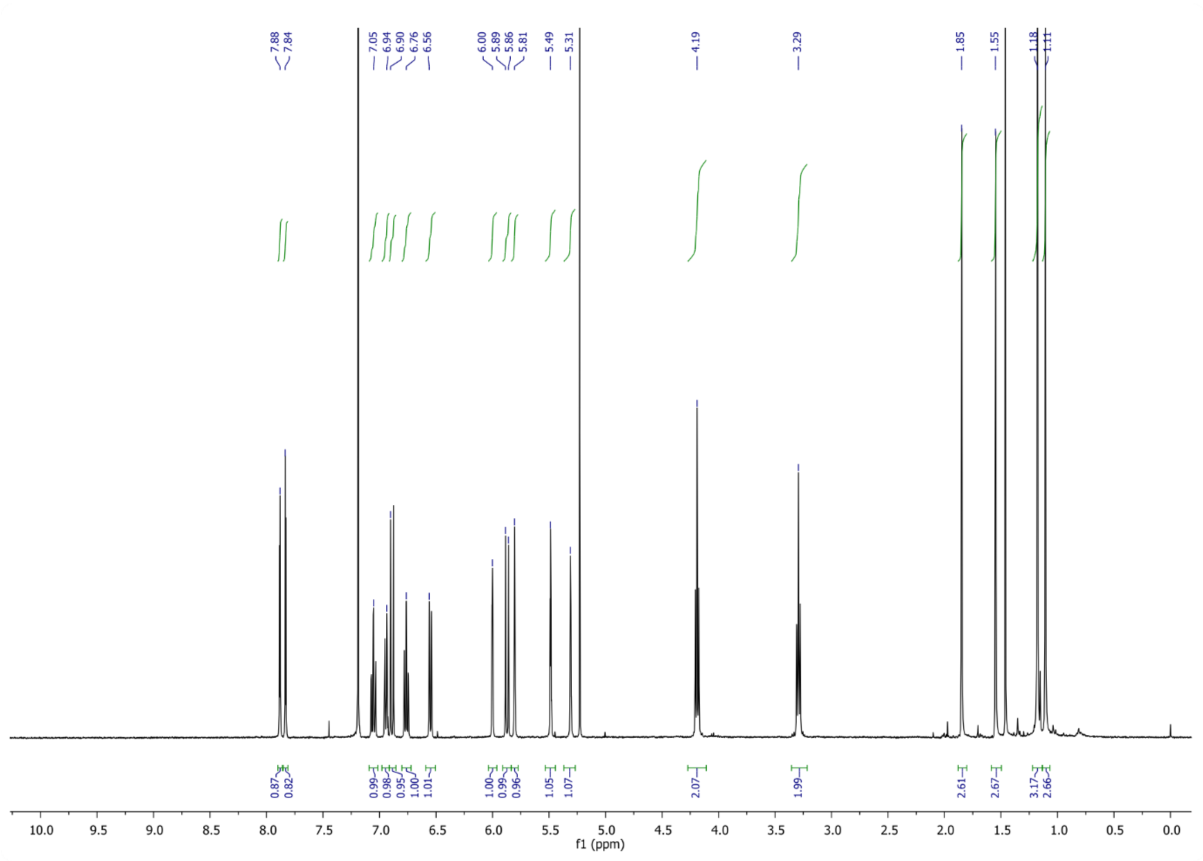

Figure S1. ^{1}H NMR spectrum of the spiropyran dimethacrylate cross-linker **2**.

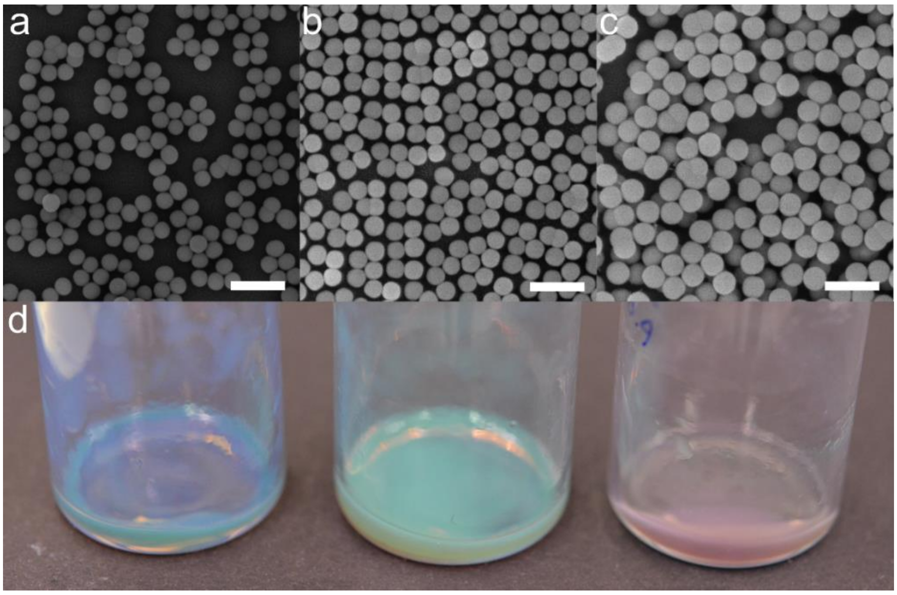

Figure S2. a-c) Scanning electron micrographs of colloidal silica particles with diameters of (a) 156 nm, (b) 163 nm, and (c) 197 nm; scale bars = 500 nm. d) Photograph of the corresponding suspensions of the particles in PEGPEA, PEGDA and HMPP that were used to prepare photonic compositions **1** to **3** from left to right.

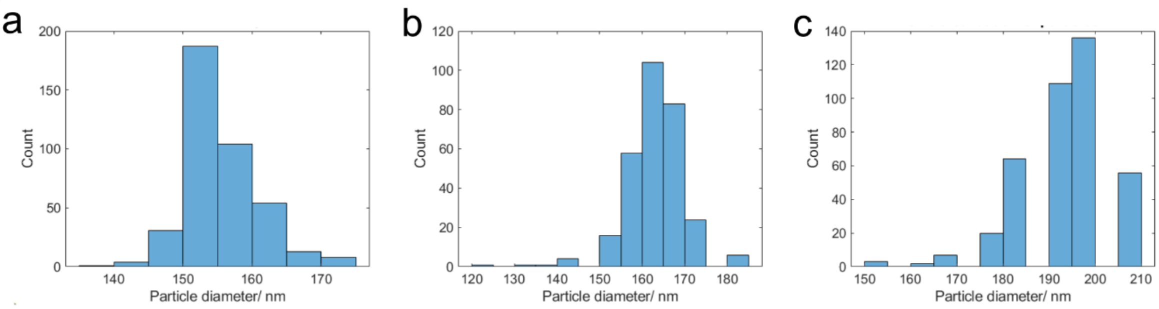

Figure S3. Size analysis of the colloidal silica particles shown in the SEM images in Figure S2.

Table S2. Size analysis of the colloidal silica particles shown in the SEM images in Figure S2. Errors in the silica particle diameter are standard errors in the mean, calculated from the analysis on three separate SEM images.

Batch number	Particle diameter/ nm	Size dispersity/ %
1	154.3 ± 3.5	4.3 ± 0.8
2	168.1 ± 4.3	4.1 ± 0.3
3	165.6 ± 4.7	4.7 ± 0.3
4	197.7 ± 3.3	5.6 ± 0.8

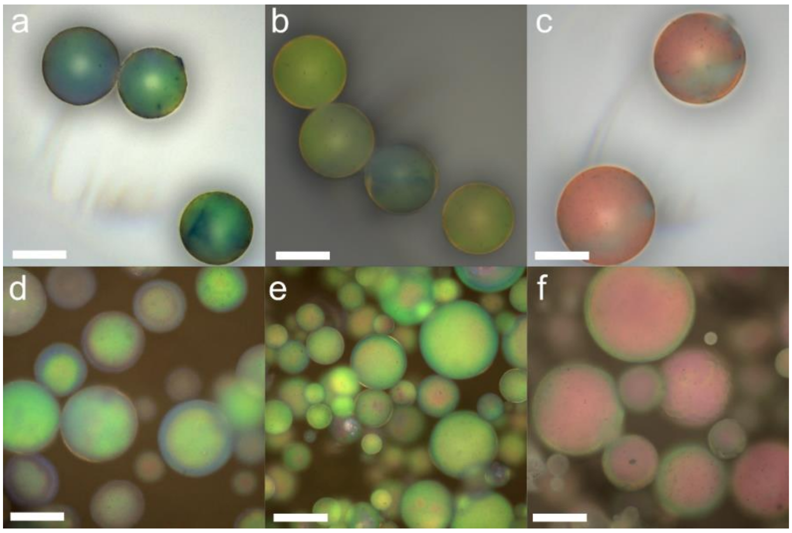

Figure S4. Optical micrographs of mechano-pigments based on photonic compositions **1** to **3** (all with an overall cross-link density of 1%) in air (a-c) and incorporated in a PDMS matrix at a concentration of 20 wt% on a dark background (d-f). Scale bars = 100 µm.

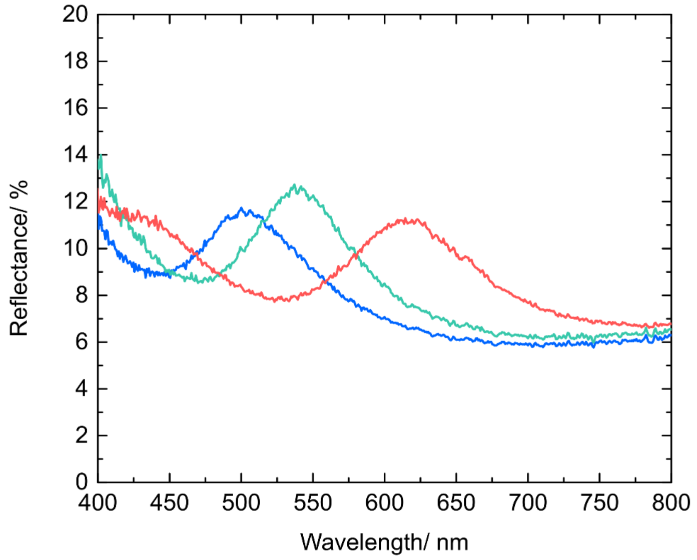

Figure S5. Reflectance spectra of the mechano-pigments shown in Figure S2 incorporated in PDMS. Each spectrum is an average of five measurements of different mechano-pigments. Photonic compositions **1** (blue), **2** (blue-green) and **3** (red).

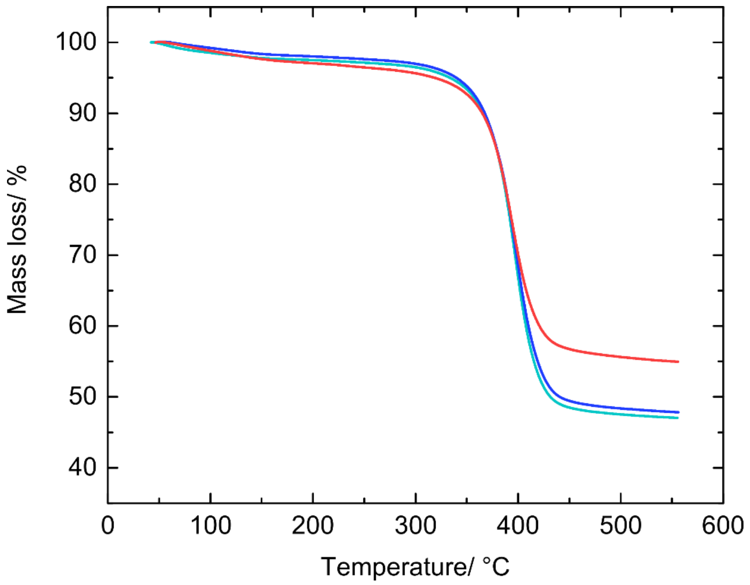

Figure S6. Thermogravimetric analysis (TGA) traces of mechano-pigments. Photonic compositions **1** (blue), **2** (blue-green) and **3** (red).

Table S3. Expected and measured volume fractions of silica, $\varphi(SiO_2)$ in three pigment batches. Calculated values from mass loss recorded in TGA measurements (Figure S6), assuming $\rho(SiO_2)$ = 2 g cm^{-3}.[3–5]

Photonic composition number	Expected $\varphi(SiO_2)$	Measured $\varphi(SiO_2)$
1	0.35	0.33
2	0.35	0.34
3	0.4	0.41

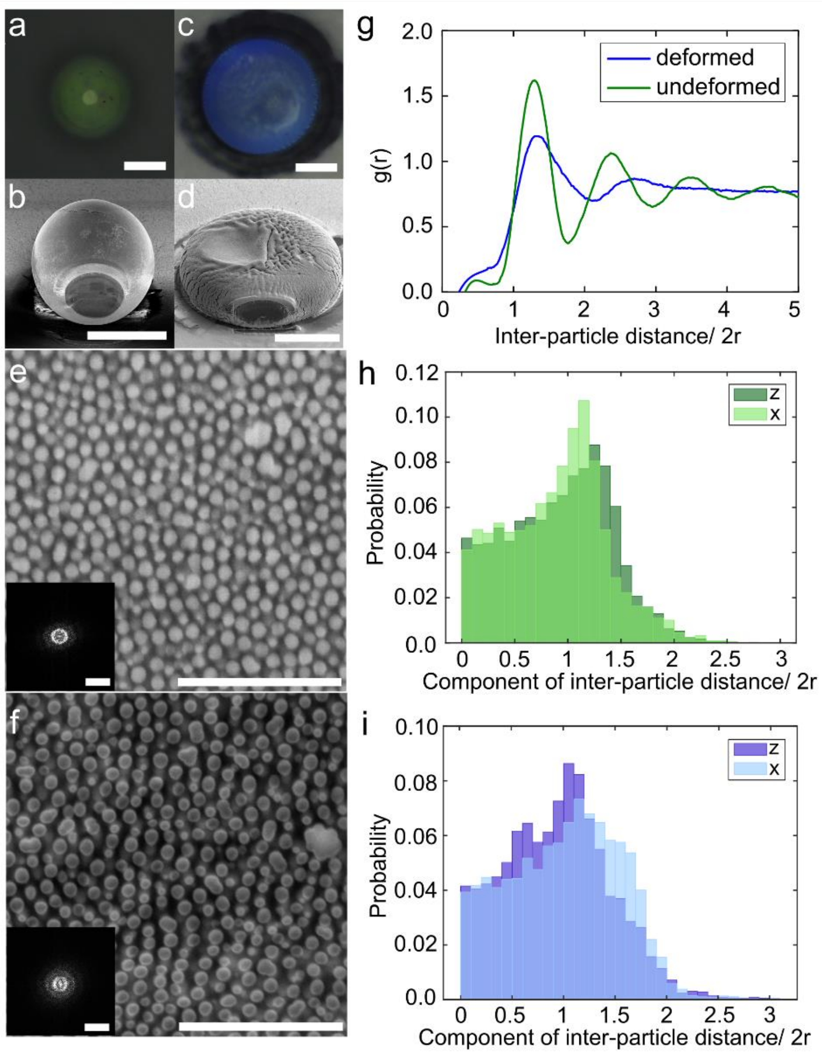

Figure S7. Deformation-induced changes of the photonic structure of the mechano-pigment, with photonic composition **2** in the absence of the spiropyran, 1 mol% cross-link density. (a) Light microscopy image of a mechano-pigment with a green photonic reflection in the as-prepared state turns (c) blue upon compression (scale bars = 50 µm); scanning electron micrographs (SEM) (b, d) show the same pigments as in (a) and (c); cuts were applied with a focused ion beam (FIB) to reveal the cross-sections (scale bars = 2 µm). (e, f) SEM images of the cross-sections of as-prepared and compressed mechano-pigments (scale bars = 2 µm). Insets show 2D fast Fourier transforms of the SEM images (scale bars = 0.0177 nm^{-1}). (g) Radial distribution function g(r) of silica colloids calculated from images shown in (e, f). The histograms of x- and z-components of the inter-colloidal distances demonstrate deformation-induced anisotropy in the colloidal arrangement of the compressed sphere (i) compared to the as-prepared state (h).

Text S1: Surface ordering of silica particles in mechano-pigments.

The surface layer of silica particles in the mechano-pigments seems to be close-packed (Figure S8-S10), which possibly results from diffusion of the PEGPEA monomer into the continuous aqueous phase in the emulsion step of the synthesis. The spectral measurements (Figure S5 and S12) indicate that the optical properties are determined by the bulk, rather than the surface layer.

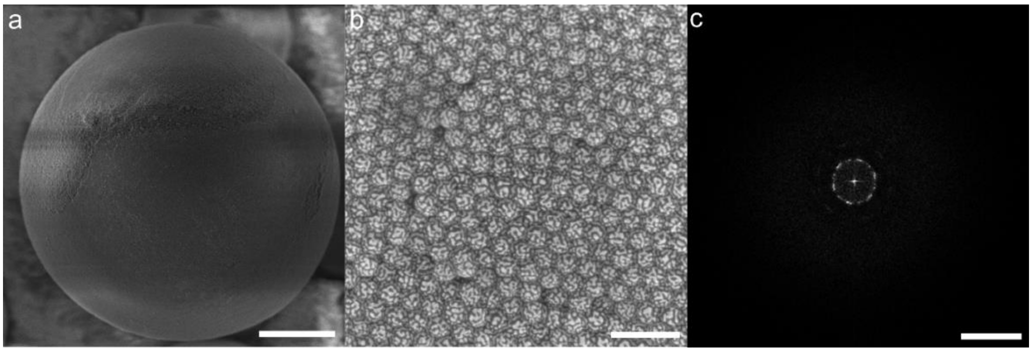

Figure S8. Electron micrographs of a spherical mechano-pigment with photonic composition **2**: a) scale bar = 20 µm, b) close-up of surface, scale bar = 500 nm. c) 2D fast Fourier transform of SEM image in b), showing a hexagonal pattern that is indicative of long-range order, scale bar = 0.0414 nm^{-1}.

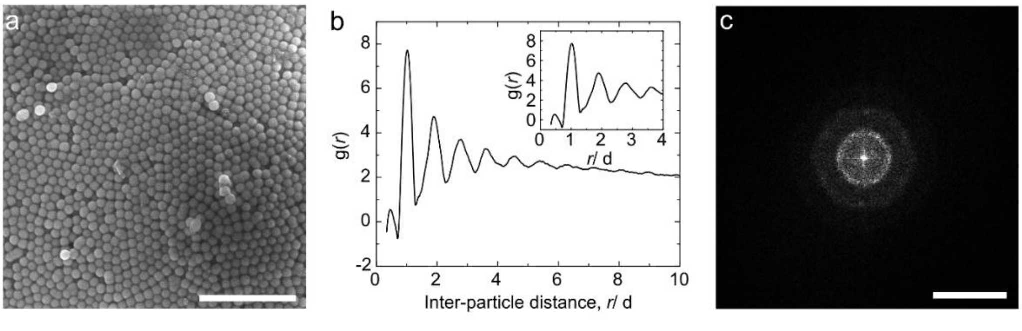

Figure S9. Characterization of the surface ordering of a mechano-pigment with photonic composition **3**: a) SEM image of surface, scale bar = 2 µm. b) Radial distribution function calculated from the SEM image in a) and inset with close-up, showing significant long-range order and close-packing (first peak occurs when inter-particle distance is equal to the colloidal diameter). c) FFT of the SEM image in a), showing a hexagonal pattern that is indicative of long-range order, scale bar = 0.0164 nm^{-1}.

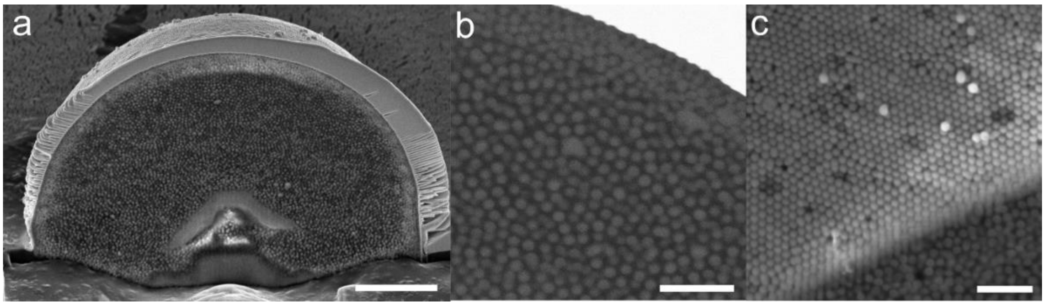

Figure S10. FIB-SEM cross-section of a spherical mechano-pigment with photonic composition **2** without spiropyran, showing a layer of more closely packed silica colloids at the surface of the sphere (note that mechano-pigment is also covered with a layer of Pt to protect sample from FIB): a) scale bar = 5 µm, b) close-up of cross-section, scale bar = 1 µm, c) close-up of edge of cross-section on another mechano-pigment without Pt layer showing both surface and interior ordering, scale bar = 1 µm.

Text S2: Calculation of expected interparticle spacing.

The predicted distance between nearest neighbors, a, defined as the distance between the centroids of two neighboring particles, was calculated assuming a face-centered cubic close-packing:[6]

$$a = \left(\frac{\pi}{3\sqrt{2}\,\varphi(\mathrm{SiO_2})}\right)^{\frac{1}{3}} d \qquad (1)$$

where d is the particle diameter and $\varphi(\mathrm{SiO_2})$ is the volume fraction of particles.

The predicted wavelength of the light most strongly reflected by the particle arrangement was calculated using Equation 2:

$$\lambda_{\max} = \left(\frac{\pi}{3\sqrt{2}\,\varphi(\mathrm{SiO_2})}\right)^{\frac{1}{3}} \left(\frac{8}{3}\right)^{\frac{1}{2}} d \qquad (2)$$

where a is the interparticle spacing and n_{eff} is the average refractive index, which is calculated as follows:

$$n_{eff}^2 = n(\mathrm{SiO_2})^2\,\varphi(\mathrm{SiO_2}) + n(\mathrm{PEGPEA})^2(1-\varphi(\mathrm{SiO_2})) \qquad (3)$$

where $n(SiO_2)$ and $n(\text{PEGPEA})$ are the refractive indices of the silica particles and the polymer matrix of the pigment, respectively.

Table S4. Average spacing between nanoparticles: predicted vs. measured from FIB-SEM images

Particle diameter, *d*/nm	Predicted interparticle spacing (Eq. S1), *a*/nm	Measured interparticle spacing, *a*/nm *	Predicted wavelength of maximum reflected intensity (Eq. S2), λ_{max}/nm	Centre of gravity of measured reflectance spectrum, λ_{max}/nm **
156.0	200.3	179.4	484.9	510
163.3	209.5	222.0	507.3	540

*From FIB-SEM; **from spectral microscope

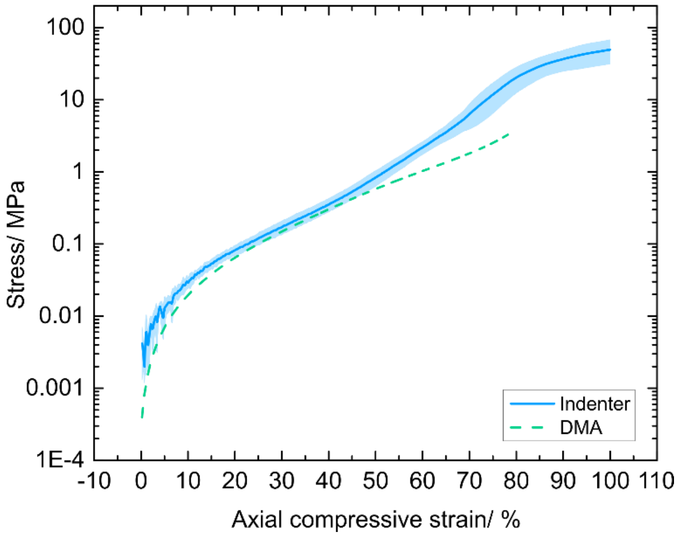

Figure S11. Stress-strain plots acquired with a custom-made compression tester (blue continuous line) and dynamic mechanical analysis in compression (DMA, green dashed line) of pigments with photonic composition **2**. Shaded area corresponds to error in average of 5 measurements. For DMA measurement, sample size was approximately 1.1 x 2.0 x 2.3 mm. For details of the sample preparation, refer to the section on the preparation of the mechano-pigments. For indenter measurements, the diameter of pigment sphere varied between 100 – 200 µm.

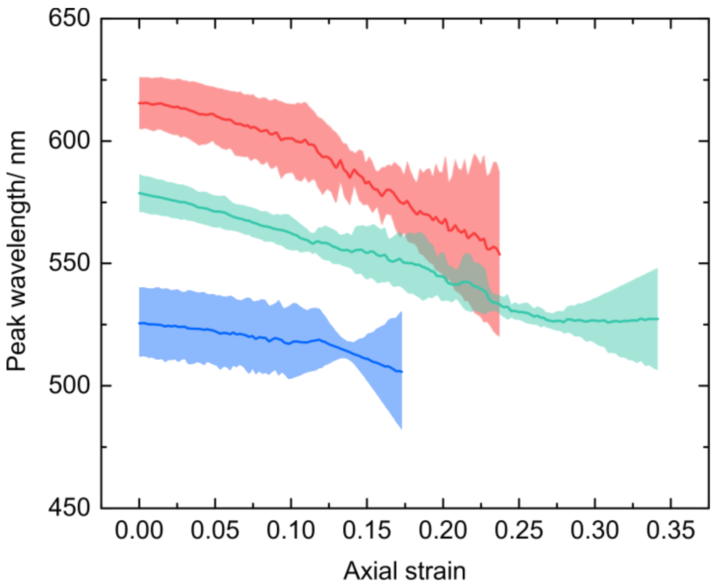

Figure S12. Change in peak wavelength with increasing axial strain in compression, measured from single mechano-pigments with different photonic compositions: **1** (blue), **2** (blue-green), **3** (red). Three separate compression experiments were conducted for each composition, with lines representing the average and shaded areas the standard deviation.

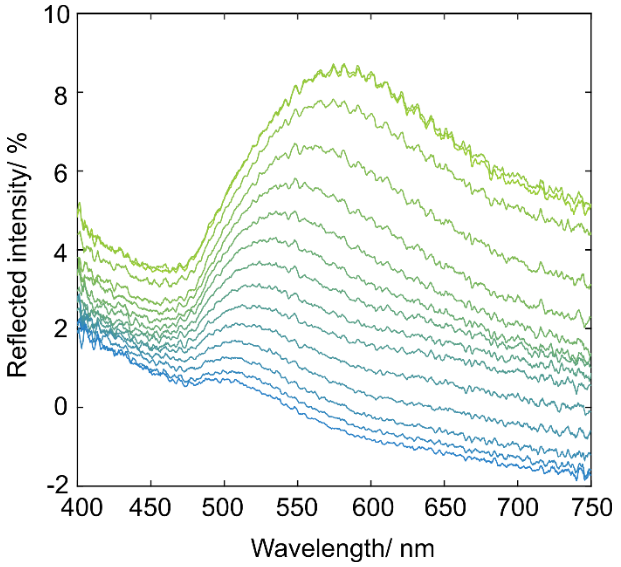

Figure S13. Raw spectra for data shown in Figure 2h, showing hypochromic shift and decrease in reflected intensity with increasing compressive strain, from 0 to 30 % axial strain. The reflectance spectra were normalized against a silver mirror. Smoothing applied with Savitsky-Golay filter (order 3, frame length 21). Pigment composition **2**.

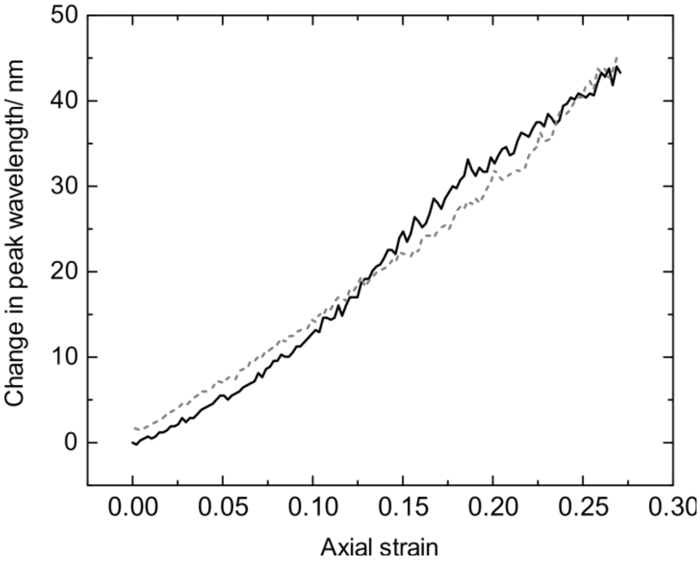

Figure S14. Spectral change in reflectance of single pigment (photonic composition **2**) in compression, showing full reversibility in one strain cycle. Black line: increasing strain; grey dashed line: decreasing strain.

Text S3: Calculation of mechano-activated fluorescence intensity.

The normalized fluorescence intensity from the merocyanine was calculated to enable a comparison between the mechano-activation in tension and in compression. Normalized fluorescence, $I_{\text{normalised}}(\varepsilon)$ was defined as:[7,8]

$$I_{\text{normalised}}(\varepsilon) = \frac{I(\varepsilon) - I(\varepsilon = 0)}{I_{\max} - I_{\min}} \tag{4}$$

where $I(\varepsilon)$ is the fluorescence intensity recorded at strain ε, $I(\varepsilon = 0)$ is the fluorescence intensity recorded at zero strain, permitting a correction for the small amount of merocyanine present in the strain-free equilibrium. $I_{\max}$ and $I_{\min}$ are the maximum (photostationary MC form) and minimum (photostationary SP form) intensities, recorded by exposing the samples to UV and green light respectively.

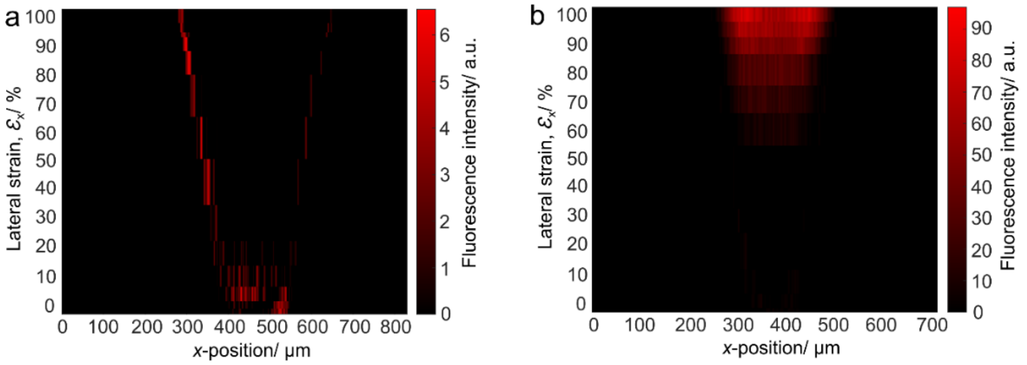

Figure S15. Normalized fluorescence intensity, determined using Equation S4, with increasing compressive strain along a central line through mechano-pigment spheres with photonic composition **2**, containing i) molecularly dissolved spiropyran and ii) covalently incorporated spiropyran, imaged with confocal microscopy, both with total cross-linking density 1 mol% and 0.25 mol% spiropyran content.

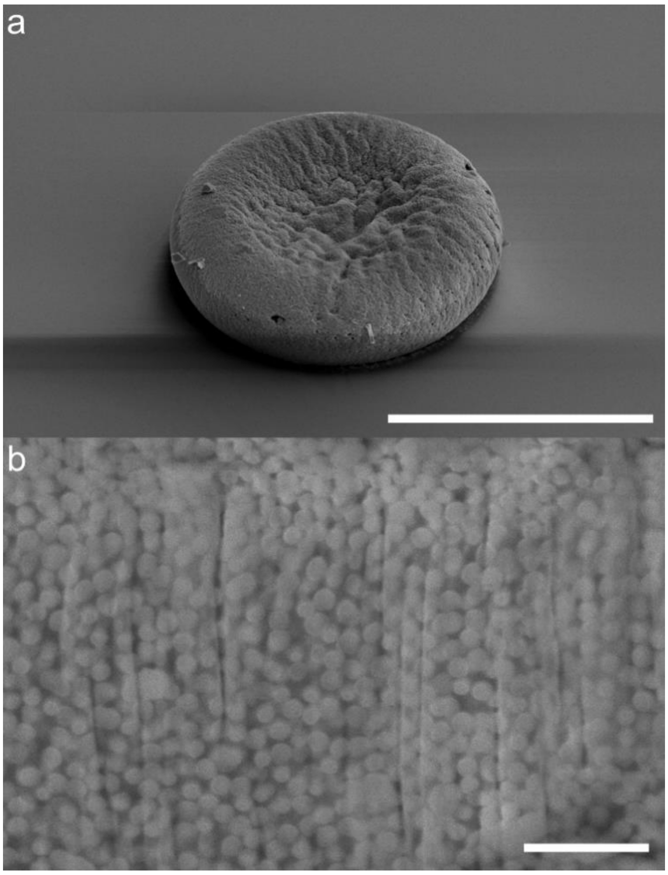

Figure S16. a) SEM images of a compressed mechano-pigment with photonic composition **2** with a collapsed central region, scale bar = 50 µm and b) cross-section at the center of the mechano-pigment, showing silica particles compressed together, scale bar = 1 µm.

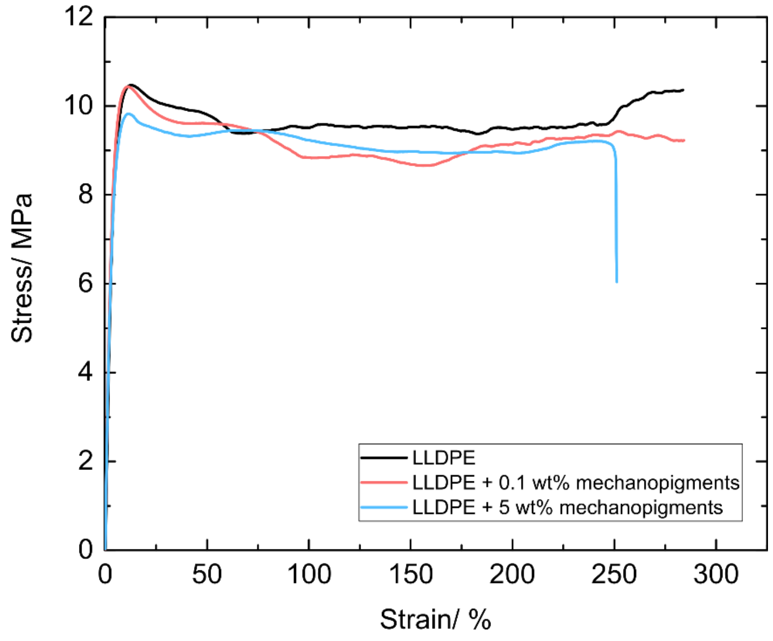

Figure S17. Stress-strain plots of linear low-density polyethylene (LLDPE) containing: no mechano-pigments (black), 0.1 wt% mechano-pigments (red), 5 wt% mechano-pigments (blue).

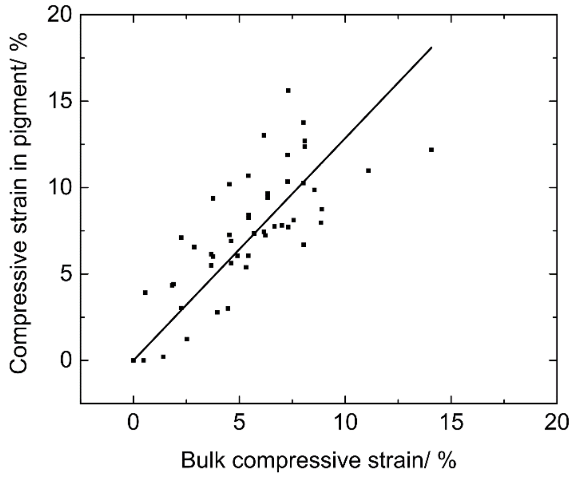

Figure S18. Compressive strain of mechano-pigments (photonic composition **2**), measured from pigment dimensions, vs. bulk compressive strain within LLDPE matrix, calculated from the applied elongational strain and assuming a Poisson ratio of 0.44.[9] The slope of the linear fit is (1.29 ± 0.059).

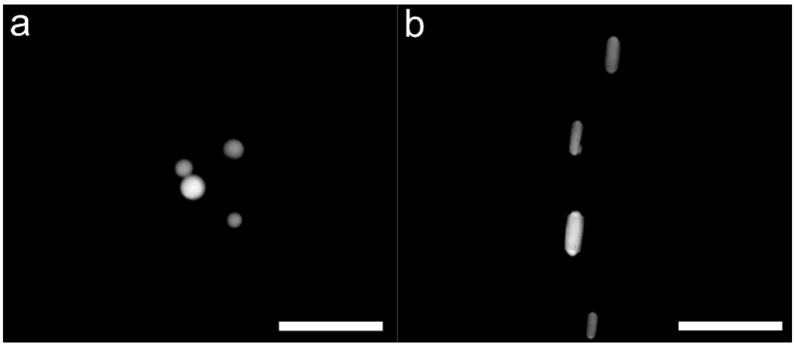

Figure S19. Fluorescence micrographs of mechano-pigments (photonic composition **2**) in which the spiropyran has been converted to fluorescent merocyanine by exposure to UV light, incorporated in LLDPE (0.33 mm thickness), a) as prepared and b) following necking. The average change in fluorescence intensity of the pigments following necking is – (7 ± 4) %. Scale bar = 500 µm.

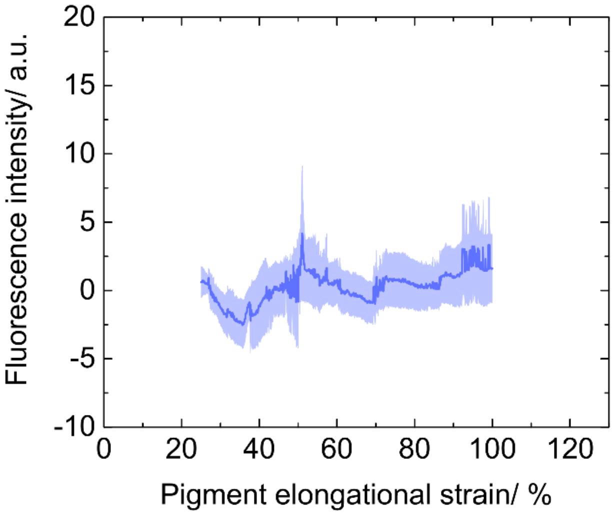

Figure S20. Fluorescence intensity (corrected for baseline intensity) of control pigments with photonic composition **2** and containing molecularly dissolved spiropyran (0.25 mol%) vs. elongational strain of pigment in LLDPE.

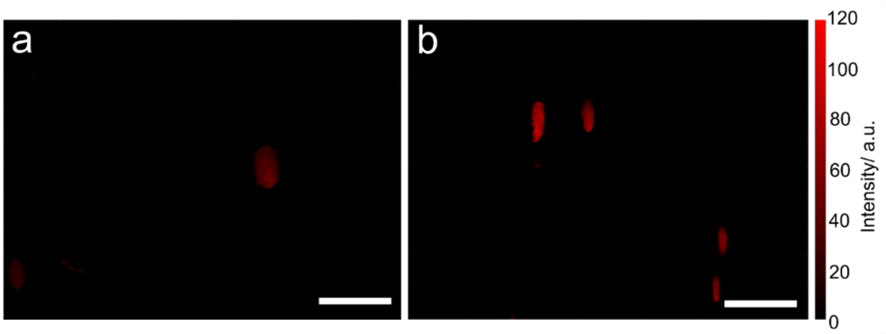

Figure S21. Fluorescence micrographs of mechano-pigments with photonic composition **2** incorporated in a single sample of LLDPE at a bulk strain of 108 %, in which the LLDPE matrix is a) not necked and b) necked. The tensile strain in the pigment is 35% in a) and 80 % in b). Scale bar = 500 µm.

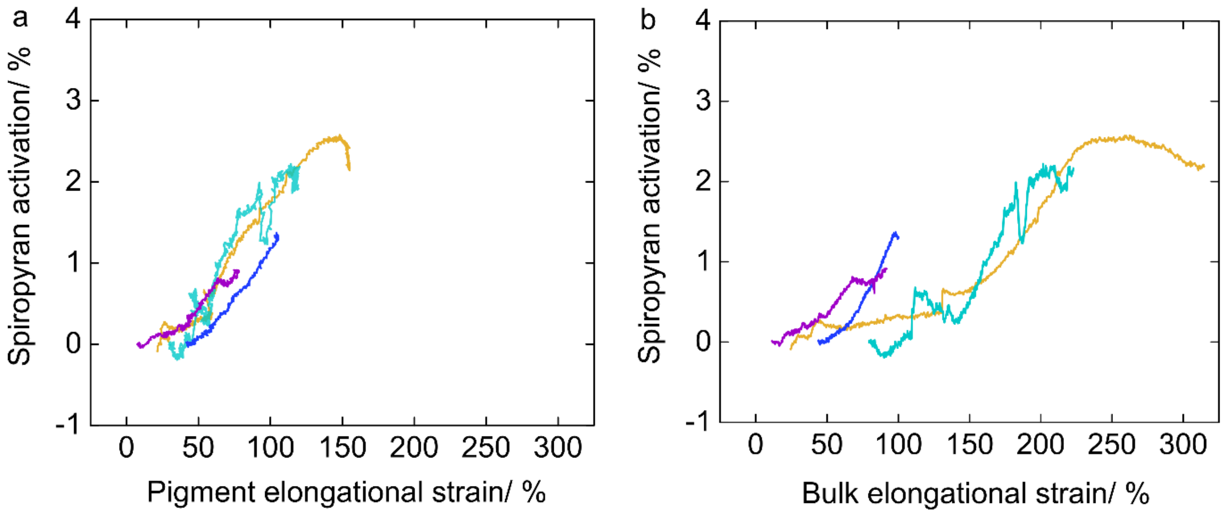

Figure S22. Mechano-activation of spiropyran in mechano-pigments with photonic composition **2** in LLDPE under tensile strain. Spiropyran activates significantly as the sphere passes into the first neck, which occurs at different ***bulk*** axial strains, depending on the location of the mechano-pigment sphere within the film. When plotted against the ***local*** strain, defined as the ratio of the change in the major axis of the pigment and the starting diameter, the mechano-activation curves collapse onto the same master curve. One of the pigments (dark yellow) shows a plateau beyond approximately 200% strain, as the neck proceeded to propagate through the rest of the sample.

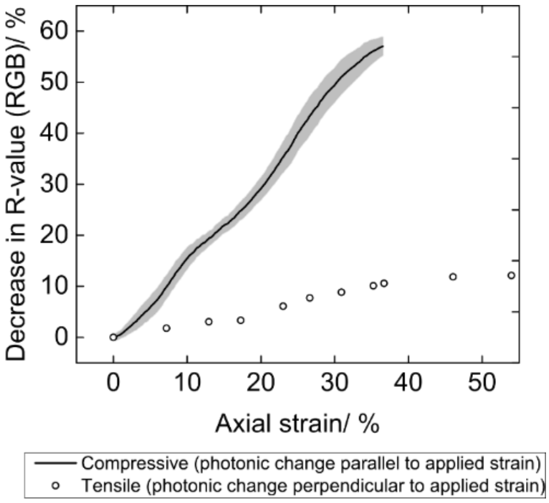

Figure S23. Photonic color change in compression (single sphere, not incorporated in a polymer matrix) and in tension (incorporated in LLDPE) vs. strain along the axis of the applied strain. In the case of compression, the periodicity decreases and a blue-shift is observed to occur along the axis of compression, whereas in the case of tension, the blue-shift is observed along the axis perpendicular to the axis of applied tensile strain.

Text S4: Indentation experiments on mechano-pigments incorporated in PDMS.

For indentation experiments, the PDMS film containing 10wt% mechano-pigments was left attached to the glass slide. A film of carbon black-containing PDMS was positioned below the film containing mechano-pigments, and the steel letter stamp ‘a’ (Figure 23a) was lightly pressed by hand into the mechano-pigment film from below, while taking a photograph from above (Figure 16b). On applying further pressure by hand, the mechano-activation of spiropyran to purple merocyanine could be observed following removal of the stamp (Figure 23c). The spectral changes upon indentation were also recorded with a spectral microscope (Figure 24). Similar colorimetric changes could also be observed upon fracturing or damaging the film with a spatula (Figure 25), and the purple mechano-activated merocyanine could furthermore by transformed back to colorless spiropyran upon exposure to green light (Figure 25c). Pigments not containing spiropyran did not show purple coloration upon indentation (Figure 26).

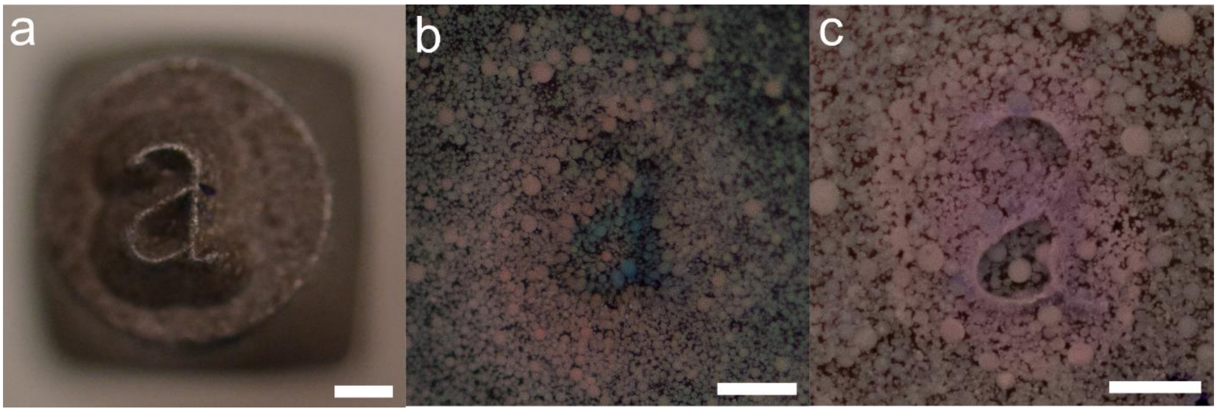

Figure S24. a) Photograph documenting the mechanochromic color changes of mechano-pigments with photonic composition **3** in a PDMS matrix upon indentation of the composite with a letter stamp (scale bar = 1 mm). b) A photonic hypsochromic shift is observed directly under indenting stamp and a bathochromic shift in the surrounding areas (scale bar = 1 mm). c) The mechano-activation of the spiropyran is reflected by purple coloration (scale bar = 1 mm).

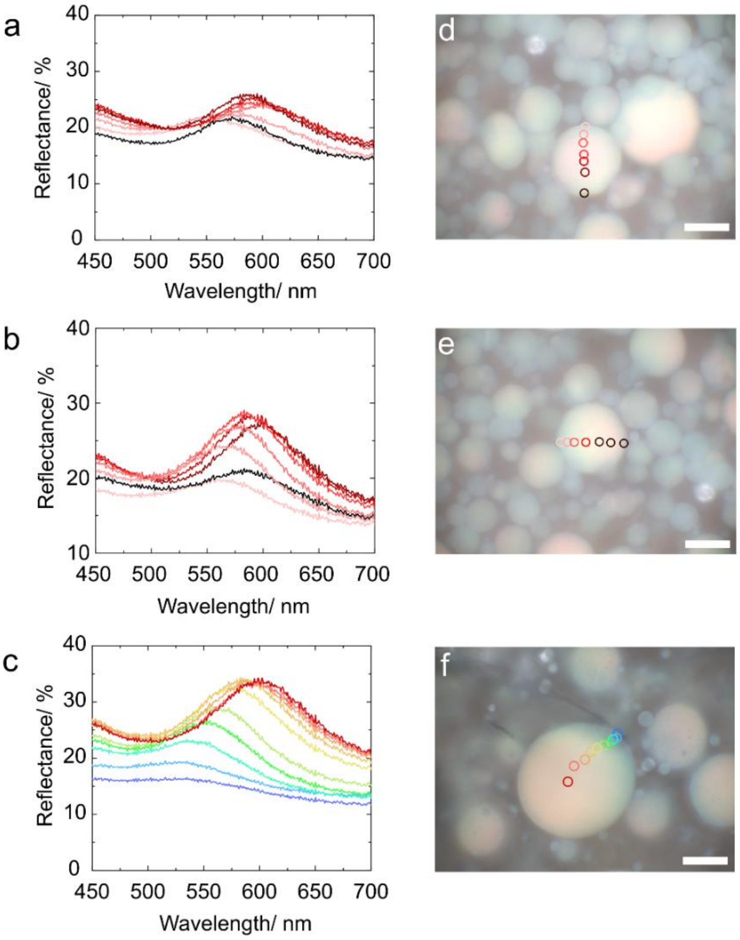

Figure S25. Spectra of pigments with photonic composition **3**, 1 mol% cross-link density and 0.25 mol% spiropyran, incorporated in PDMS, at spots marked by circles in micrographs (scale bar = 100 µm), in unperturbed pigments (spectra: a,b; micrographs: d,e) and a compressed pigment showing a hypsochromic spectral shift towards the site of indentation (spectra: c; micrographs: f).

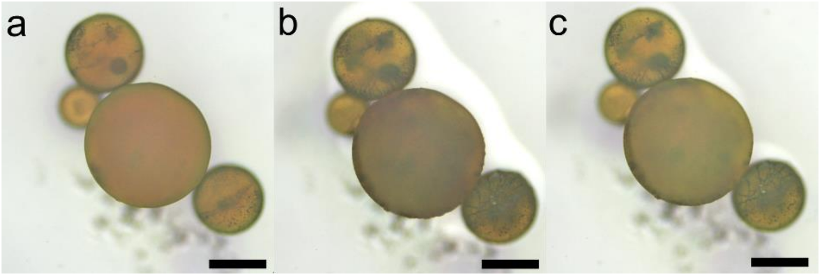

Figure S26. Micrographs of mechano-pigments with photonic composition **3**, 1 mol% cross-link density and 0.25 mol% spiropyran, incorporated in PDMS, a) before compression, b) after compression, showing purple coloration from mechano-activated merocyanine and c) after compression and green light exposure to revert merocyanine to spiropyran. The photonic component remains green, indicating that it has not fully relaxed to its starting state as a result of permanent deformation to the pigments and the PDMS matrix. Scale bars = 200 µm.

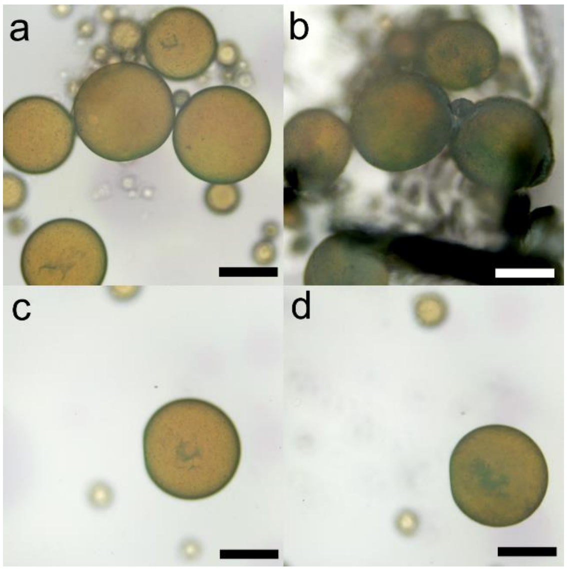

Figure S27. Micrographs of mechano-pigment spheres with photonic composition **3**, 1 mol% cross-link density without spiropyran, incorporated in PDMS, a) and c) before compression, b) and d) after compression. Scale bars = 200 µm.

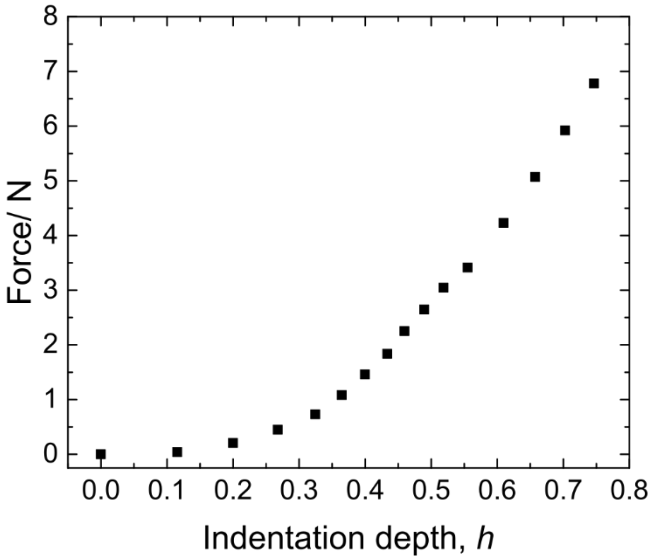

Figure S28. Force vs. indentation depth for PDMS containing 10 wt% mechano-pigments, where indentation depth is defined as indentation distance/ initial film thickness.

References

[1] D. A. Davis, A. Hamilton, J. Yang, L. D. Cremar, D. Van Gough, S. L. Potisek, M. T. Ong, P. V. Braun, T. J. Martínez, S. R. White, J. S. Moore, N. R. Sottos, *Nature* **2009**, *459*, 68.

[2] G. R. Gossweiler, G. B. Hewage, G. Soriano, Q. Wang, G. W. Welshofer, X. Zhao, S. L. Craig, *ACS Macro Lett.* **2014**, *3*, 216.

[3] A. K. Van Helden, J. W. Jansen, A. Vrij, *J. Colloid Interface Sci.* **1981**, *81*, 354.

[4] A. Vrij, J. W. Jansen, J. K. G. Dhont, C. Pathmamanoharan, M. M. Kops-Werkhoven, H. M. Fijnaut, *Faraday Discuss. Chem. Soc.* **1983**, *76*, 19.

[5] C. Pathmamanoharan, *Colloids and Surfaces* **1988**, *34*, 81.

[6] G. H. Lee, T. M. Choi, B. Kim, S. H. Han, J. M. Lee, S. H. Kim, *ACS Nano* **2017**, *11*, 11350.

[7] C. M. Kingsbury, P. A. May, D. A. Davis, S. R. White, J. S. Moore, N. R. Sottos, *J. Mater. Chem.* **2011**, *21*, 8381.

[8] C. M. Degen, P. A. May, J. S. Moore, S. R. White, N. R. Sottos, *Macromolecules* **2013**, *46*, 8917.

[9] A. Dorigato, A. Pegoretti, J. Kolarik, *Polym. Polym. Compos.* **2010**, *31*, 1947.
